# Mendelian adult-onset leukodystrophy genes in Alzheimer's disease: critical influence of *CSF1R* and *NOTCH3*

**DOI:** 10.1016/j.neurobiolaging.2018.01.015

**Published:** 2018-06

**Authors:** Celeste Sassi, Michael A. Nalls, Perry G. Ridge, Jesse R. Gibbs, Michelle K. Lupton, Claire Troakes, Katie Lunnon, Safa Al-Sarraj, Kristelle S. Brown, Christopher Medway, Jenny Lord, James Turton, Jose Bras, ARUK Consortium, Peter Passmore, David Craig, Janet Johnston, Bernadette McGuinness, Stephen Todd, Reinhard Heun, Heike Kölsch, Patrick G. Kehoe, Emma R.L.C. Vardy, Nigel M. Hooper, David M. Mann, Stuart Pickering-Brown, Kristelle Brown, James Lowe, Kevin Morgan, A. David Smith, Gordon Wilcock, Donald Warden, Clive Holmes, Sonja Blumenau, Mareike Thielke, Christa Josties, Dorette Freyer, Annette Dietrich, Monia Hammer, Michael Baier, Ulrich Dirnagl, Kevin Morgan, John F. Powell, John S. Kauwe, Carlos Cruchaga, Alison M. Goate, Andrew B. Singleton, Rita Guerreiro, Angela Hodges, John Hardy

**Affiliations:** a Reta Lila, Weston Research Laboratories, Department of Molecular Neuroscience, UCL Institute of Neurology, London, UK; b Laboratory of Neurogenetics, National Institute on Aging, National Institutes of Health, Bethesda, MD, USA; c Department of Experimental Neurology, Center for Stroke Research Berlin (CSB), Charité – Universitätsmedizin Berlin, Corporate Member of Freie Universität Berlin, Humboldt-Universität zu Berlin, and Berlin Institute of Health, Berlin, Germany; d Departments of Biology, Neuroscience, Brigham Young University, Provo, UT, USA; e King's College London Institute of Psychiatry, London, UK; f QIMR Berghofer Medical Research Institute, Brisbane, Queensland, Australia; g Institute of Biomedical and Clinical Science, University of Exeter Medical School, Exeter, Devon, UK; h Translation Cell Sciences-Human Genetics, School of Life Sciences, Queens Medical Centre, University of Nottingham, Nottingham, UK; i Department of Molecular Neuroscience, Institute of Neurology, University College London, London, UK; j Department of Medical Sciences, Institute of Biomedicine-iBiMED, University of Aveiro, Aveiro, Portugal; k UK Dementia Research Institute at UCL (UK DRI), London, UK; l Neurodegenerative Diseases, Robert-Koch-Institut, Berlin, Germany; m Department of Neuroscience, Brigham Young University, Provo, UT, USA; n Division of Biology and Biomedical Sciences, Washington University, St. Louis, MO, USA; o Icahn School of Medicine at Mount Sinai, Icahn Medical Institute, New York, NY, USA

**Keywords:** Alzheimer's disease, Mendelian leukodystrophies, *CSF1R*, *NOTCH3*

## Abstract

Mendelian adult-onset leukodystrophies are a spectrum of rare inherited progressive neurodegenerative disorders affecting the white matter of the central nervous system. Among these, cerebral autosomal dominant and recessive arteriopathy with subcortical infarcts and leukoencephalopathy, cerebroretinal vasculopathy, metachromatic leukodystrophy, hereditary diffuse leukoencephalopathy with spheroids, and vanishing white matter disease present with rapidly progressive dementia as dominant feature and are caused by mutations in *NOTCH3*, *HTRA1*, *TREX1*, *ARSA*, *CSF1R*, *EIF2B1*, *EIF2B2*, *EIF2B3*, *EIF2B4*, and *EIF2B5,* respectively. Given the rare incidence of these disorders and the lack of unequivocally diagnostic features, leukodystrophies are frequently misdiagnosed with common sporadic dementing diseases such as Alzheimer's disease (AD), raising the question of whether these overlapping phenotypes may be explained by shared genetic risk factors. To investigate this intriguing hypothesis, we have combined gene expression analysis (1) in 6 different AD mouse strains (APPPS1, HOTASTPM, HETASTPM, TPM, TAS10, and TAU) at 5 different developmental stages (embryo [E15], 2, 4, 8, and 18 months), (2) in APPPS1 primary cortical neurons under stress conditions (oxygen-glucose deprivation) and single-variant–based and single-gene–based (c-alpha test and sequence kernel association test (SKAT)) genetic screening in a cohort composed of 332 Caucasian late-onset AD patients and 676 Caucasian elderly controls. *Csf1r* was significantly overexpressed (log2FC > 1, adj. *p*-value < 0.05) in the cortex and hippocampus of aged HOTASTPM mice with extensive Aβ dense-core plaque pathology. We identified 3 likely pathogenic mutations in *CSF1R* TK domain (p.L868R, p.Q691H, and p.H703Y) in our discovery and validation cohort, composed of 465 AD and mild cognitive impairment (MCI) Caucasian patients from the United Kingdom. Moreover, *NOTCH3* was a significant hit in the c-alpha test (adj *p*-value = 0.01). Adult-onset Mendelian leukodystrophy genes are not common factors implicated in AD. Nevertheless, our study suggests a potential pathogenic link between *NOTCH3*, *CSF1R*, and sporadic late-onset AD, which warrants further investigation.

## 1 Introduction

Mendelian adult-onset leukodystrophies are a spectrum of rare chronic progressive disorders affecting the white matter of the central nervous system. Although a growing body of literature is reporting newly discovered forms, the most characterized adult-onset leukodystrophies are cerebral autosomal dominant and recessive arteriopathy with subcortical infarcts and leukoencephalopathy (CADASIL and CARASIL), cerebroretinal vasculopathy (CRV), metachromatic leukodystrophy (MLD), hereditary diffuse leukoencephalopathy with spheroids (HDLS) and vanishing white matter disease (VWM), caused by mutations in *NOTCH3*, *HTRA1*, *TREX1*, *ARSA*, *CSF1R*, *EIF2B1*, *EIF2B2*, *EIF2B3*, *EIF2B4*, *EIF2B5,* respectively (Joutel et al., 1996), (Hara et al., 2009), (Richards et al., 2007), (Fluharty et al., 1991), (Rademakers et al., 2011), (Scali et al., 2006). Given the rare incidence of these disorders (5/100,000 to only few cases reported), the lack of peculiar and distinctive (1) clinical features, generally represented by rapidly progressive dementia, behavioral changes, pyramidal and extrapyramidal signs and, less commonly, ischemic strokes and epileptic seizures; (2) magnetic resonance imaging (MRI) lesion patterns, normally characterized by T2-weighted periventricular and subcortical, patchy and later confluent white matter hyperintensities with prominent frontal involvement; and (3) neuropathological features, frequently a combination of diverse neurodegenerative hallmarks, these rare Mendelian disorders are most frequently underrecognized and misdiagnosed with common sporadic dementias such as Alzheimer's disease (AD). On the other hand, motor features like ataxia and spasticity may appear in the course of AD progression, particularly in the cases caused by or associated to *PSEN1* mutations (Rossor et al., 2010) and AD patients may display MRI patterns and neuropathological features typical of adult-onset leukodystrophies (Smith et al., 2000, Marnane et al., 2016, Barber et al., 1999, Guerreiro et al., 2013), suggesting a potential common pathogenic ground.

In the past 10 years, next-generation sequencing (NGS) paved the way for groundbreaking discoveries in AD, showing that Mendelian rare disorders offer a unique window into the sporadic complex traits and, particularly, that rare alleles in *TREM2*, *TYROBP*, and *NOTCH3*, causative for adult-onset leukodystrophies, significantly influence the susceptibility for AD (Guerreiro et al., 2012, Guerreiro et al., 2013, Ma et al., 2015). Moreover, the sequencing of different mouse strains showed extensive similarities between mouse and human genome and validated the importance of using mouse models to illuminate the genetics of human diseases (Cheng et al., 2014, Yue et al., 2014). Nevertheless, NGS still presents 2 main challenges: (1) the huge amount of data generated is difficult to mine and (2) the investigation of rare coding variants requires several thousands of samples. Consequently, the need for experimental methods that accurately identify critical genes and strategies to empower association studies became priorities. Therefore, we have applied a combination of cortical and hippocampal gene expression analysis in 6 diverse AD mouse strains (APPPS1, HOTASTPM, HETASTPM, TPM, TAS10, and TAU) at 5 different developmental stages (embryo [E15], 2, 4, 8 and 18 months) to comprehensively study leukodystrophy gene expression pattern in relation to the progression of AD neuropathology and under stress conditions such as oxygen-glucose deprivation (OGD), which represents an *in vitro* model of ischemic stroke, a common feature in several adult-onset leukodystrophies and frequent comorbidity in AD. We then used exome and genome sequencing data in a cohort composed of 332 Caucasian late-onset AD (LOAD) patients and 676 Caucasian elderly controls to investigate rare coding variability in these main adult-onset Mendelian leukodystrophy genes. Among the studied genes, *Csf1r* was the only gene significantly overexpressed (log2FC>1, *p*-value < 0.05) in AD mouse models and its expression tightly correlated with the severity of dense-core plaque deposition. Moreover, we identified a total of 3 rare variants in *CSF1R* tyrosine kinase (TK) domain and TK flanking regions (p.L868R and p.D565N, p.G957R, respectively) present only in cases and very likely pathogenic. We then screened *CSF1R* in an independent cohort composed of 465 mild cognitive impairment (MCI) and AD cases, identifying 2 additional mutations in *CSF1R* TK domain (p.Q691H and p.H703Y). Finally, *NOTCH3* was a significant hit in the gene-based analysis (adj *p*-value = 0.01), suggesting a potential role as a disease modifier. We conclude that rare coding variability in adult-onset Mendelian leukodystrophy genes is not a common risk factor for AD. However, *CSF1R* coding variants clustering in the TK domain and *NOTCH3* may influence AD susceptibility.

## 2 Materials and methods

### 2.1 Adult-onset leukodystrophy gene selection

The selected genes are all Mendelian leukodystrophy causative genes with a core clinical hallmark represented by adult-onset subacute dementia with frontal predominance revealed by T2-weighted MRI (Supplementary Table S1). Moreover, all of these candidate genes present more than one of the following features: (1) previously reported misdiagnosis with AD (CADASIL, HDLS, CRV, MLD) (Guerreiro et al., 2012, Johannsen et al., 2001, Rademakers et al., 2011, Richards et al., 2007); (2) molecular interaction with other genes playing a key role in AD (*NOTCH3* and *CSF1R*) (Otero et al., 2009, Thijs et al., 2003); (3) genes taking part to APP-amyloid beta (Aβ) metabolism (*NOTCH3* and *HTRA1*) (Grau et al., 2005); (4) copresence of AD neuropathological hallmarks reported (*NOTCH3* and *CSF1R*) (Baba et al., 2006, Paquet et al., 2010); and (5) most frequently mutated genes in adults with leukoencephalopathies (*NOTCH3*, *EIF2B4, EIF2B5*, and *CSF1R*) (Lynch et al., 2017).

**Fig. 1:**
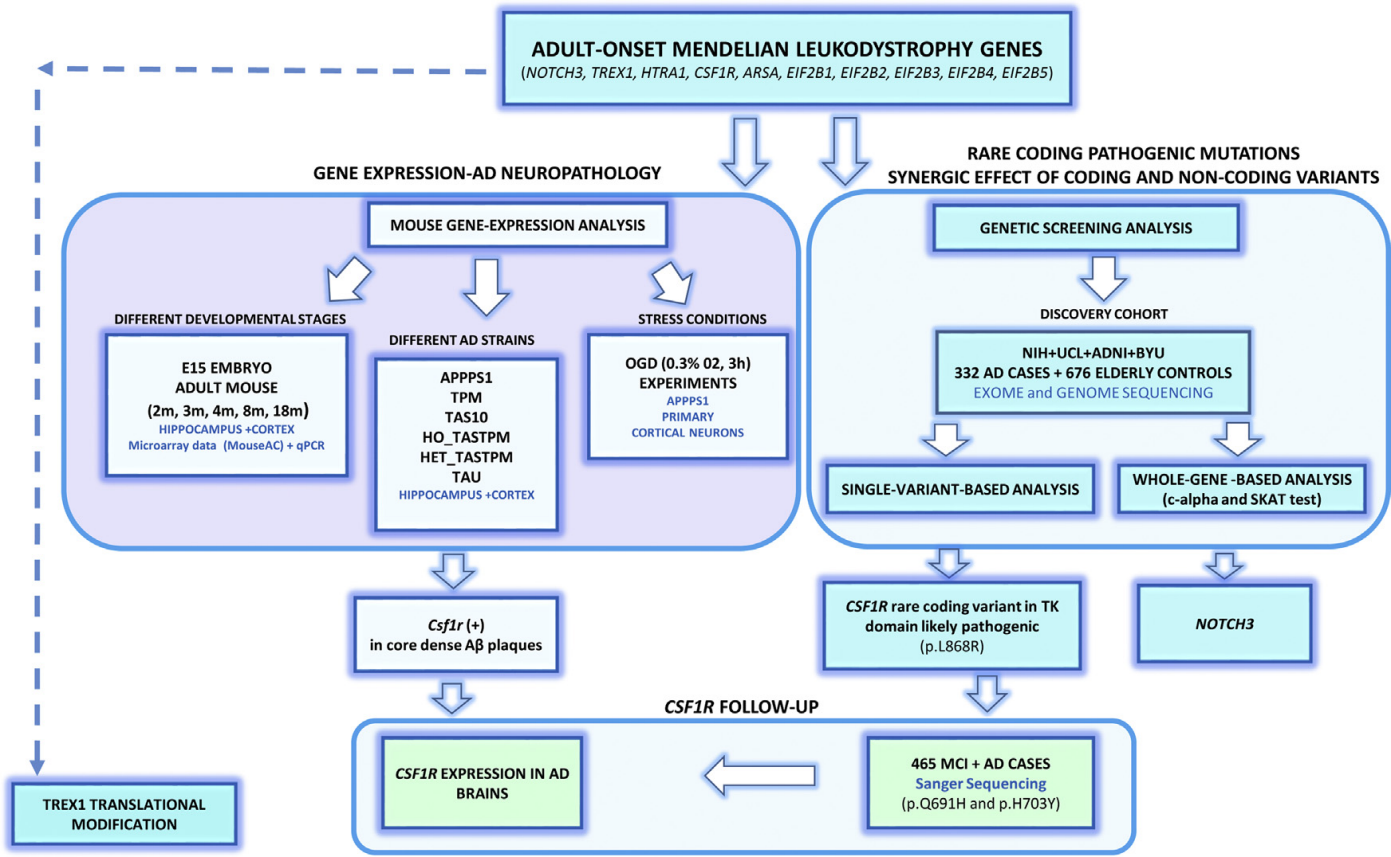
Pipeline followed in the adult-onset leukodystrophy gene study. Abbreviations: AD, Alzheimer's disease; BYU, Brigham Young University; MCI, mild cognitive impairment; TK, tyrosine kinase; NIH, National Institutes of Health; UCL, University College London.

The pipeline followed in this study is described in Fig. 1.

### 2.2 Gene expression analysis

**Supplementary Fig. S1:**
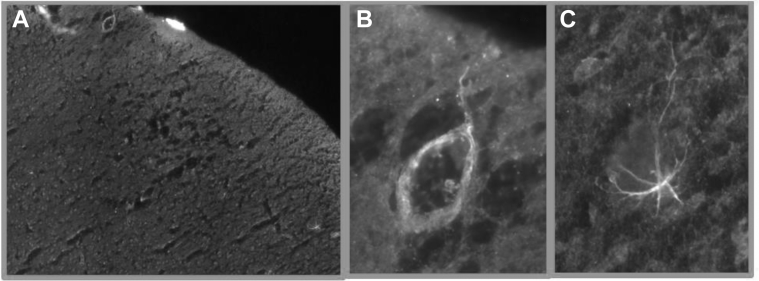
(A) Histological cortical section of APPPS1 mouse, 2 months of age, stained with Aβ oligomers (Ab126892). Rare Aβ oligomers are detected around small cortical vessels (B) and in the extracellular space, likely phagocytated by astrocytes (C), showing a very mild pathology and disease state. No Aβ plaques have been detected (data not shown).

We have used microarray data publicly available (MouseAC database [http://www.mouseac.org/]) (Matarin et al., 2015) and real-time polymerase chain reaction (RT-PCR) data to analyze *Arsa*, *Csf1r*, *Eif2b1*, *Eif2b2*, *Eif2b3*, *Eif2b4*, *Eif2b5*, *Htra1*, *Notch3*, and *Trex1* gene expression (1) in the hippocampus and cortex of 6 different AD mouse strains (APPPS1, HOTASTPM, HETASTPM, TPM, TAS10, and TAU); (2) at 5 different time points (E15, 2, 4, 8, and 18 months), to comprehensively follow expression changes related to Aβ plaque density (HOTASTPM, HETASTPM, and TAS10), neurofibrillary tangles (TAU) and absence of pathology (E15 and TPM). Adult APPPS1 data for hippocampus were available only for 2 months of age, where no plaques were reported but only rare Aβ oligomers in the cortex and surrounding cortical vessels (Supplementary Fig. S1). Finally, considering that ischemic stroke is a common feature in several leukodystrophies and frequent comorbidity in AD, we used an *in vitro* model of ischemic stroke and performed OGD experiments in APPPS1 primary cortical neurons to test whether leukodystrophy gene expression pattern may have significantly differed between APPPS1 and wild-type (WT) mice under stress conditions.

### 2.3 Genetic screening

#### 2.3.1 The discovery cohort

The discovery cohort was composed of 332 apparently sporadic AD cases and 676 elderly controls, neuropathologically and clinically confirmed, originating from the United Kingdom and North America. The mean age at disease onset was 71.66 years (range 41–94 years) for cases and the mean age of ascertainment was 78.15 years (range 60–102 years) for controls. Most of the cases (77%) were late-onset (>65 years at onset). Among the cases and controls, 42% and 51% were females, respectively; 58% and 47% of the cases and controls carried the *APOE* ε4 allele, respectively. The *APOE* ε4 allele was significantly associated to the disease status in the National Institutes of Health (NIH) and Alzheimer's Disease Neuroimaging Initiative (ADNI) series (*p*-value = 0.02 and 1.19 × 10E−9, respectively). This cohort has already been described elsewhere (Sassi et al., 2016). The threshold call rate for inclusion of the subject in analysis was 95%. On this cohort, we performed (1) gene-based analysis (SKAT and c-alpha tests) and (2) single-variant association analysis. Finally, we followed up, in an independent Caucasian data set, *CSF1R*, the only gene significantly overexpressed during AD most severe pathology (Fig. 1, Supplementary Table S2).

#### 2.3.2 The follow-up data set

The follow-up data set was composed of 296 AD and 169 MCI late-onset cases (mean age at onset >75 years) from the United Kingdom (Supplementary Table S2). Written informed consent was obtained for each clinically assessed individual, and the study was approved by the appropriate institutional review boards. All samples had fully informed consent for retrieval and were authorized for ethically approved scientific investigation (UCLH Research Ethics Committee number 10/H0716/3, BYU IRB, Cardiff REC for Wales 08/MRE09/38+5, REC Reference 04/Q2404/130, National Research Ethics Service).

#### 2.3.3 Exome and genome sequencing

DNA was extracted from blood or brain for cases and brain only for controls using standard protocols. Library preparation for NGS was based on Roche Nimblegen Inc. or TruSeq, Illumina protocols and has been described elsewhere (Sassi et al., 2016). Genome sequencing was performed in 199 controls, from the Cache County Study on Memory in Aging. All samples were sequenced with the use of Illumina HiSeq technology.

Sequence alignment and variant calling were performed against the reference human genome (UCSC hg19) and has been described in the Supplementary materials and methods.

Initial analysis excluded pathogenic mutations in *APP*, *PSEN1*, *PSEN2*, *MAPT*, *GRN*, and *TREM2*. All variants within the coding regions of the 10 adult-onset leukodystrophy candidate genes (*ARSA* [NM_000487]; *CSF1R* [NM_005211]; *EIF2B1* [NM_001414]; *EIF2B2* [NM_014239]; *EIF2B3* [NM_001261418]; *EIF2B4* [NM_001034116]; *EIF2B5* [NM_003907]; *HTRA1* [NM_002775]; *NOTCH3* [NM_000435], and *TREX1* [NM_016381] have been collected and analyzed, including 20.8 Megabase pairs of coding sequence.

#### 2.3.4 Sanger sequencing

Mutations in *CSF1R* TK domain and flanking regions were validated with Sanger Sequencing*. CSF1R* was screened in an additional follow-up cohort composed of 296 AD and 169 MCI cases (Supplementary Materials and Methods).

### 2.4 Statistical analysis

In the single-variant analysis, allele frequencies were calculated for each low-frequency and rare coding variant in cases and controls, and Fisher's exact test on allelic association was performed. MouseAC data have been analyzed and false discovery rate (FDR) correction was applied.

The Supplementary data provide a more detailed description of the methods used (mouse and human gene expression analysis, OGD experiments, Sanger sequencing, statistical analysis, and bioinformatics).

## 3 Results

### 3.1 Gene expression analysis

**Fig. 2:**
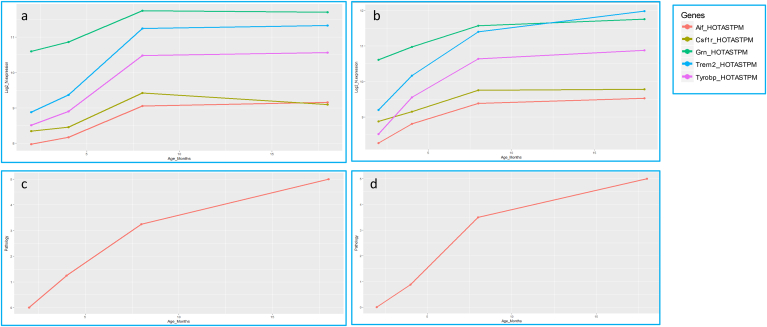
Log2-normalized expression of *Csf1r*, *Grn*, *Trem2*, *Tyrobp*, and *Aif1* in HOTASTPM mice and related Aβ plaque pathology. (A–B) Log2-normalized expression of *Csf1r*, *Grn*, *Trem2*, *Tyrobp* and *Aif1* in HOTASTPM mice (homozygous for the Swedish mutation *APP* p.K670N/M671L and *PSEN1* p.M146V) at 4 different time points (2, 4, 8, and 18 months) in the hippocampus (A) and cortex (B) showing coexpression of the above genes. (C–D) Progression of AD pathology in hippocampus (C) and cortex (D), based on Aβ plaque density, in HOTASTPM mice at 4 different time points (2, 4, 8, and 18 months). Significant *Csf1r*, *Grn*, *Trem2*, *Tyrobp*, and *Aif1* overexpression (Log2FC > 1, adj. p-value < 0.05) is detected at 8 months, in linear correlation with the most rapid and severe Aβ plaque deposition. Abbreviations: Aβ, amyloid beta; AD, Alzheimer's disease.

**Fig. 3:**
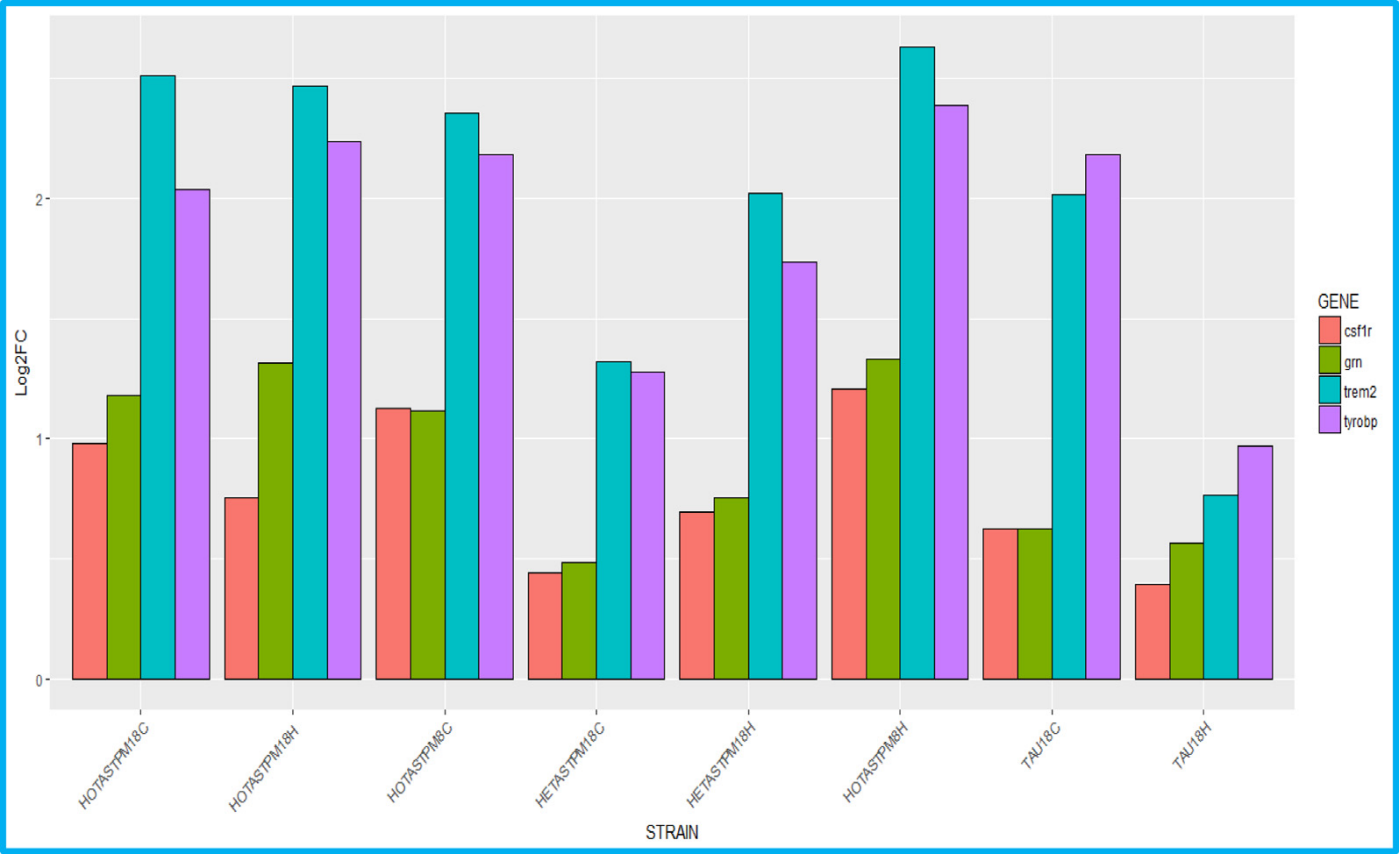
Log2FC of *Csf1r*, *Grn*, *Trem2*, and *Tyrobp* in different AD mouse strains during the most severe pathology, showing coexpression of *Csf1r*, *Grn*, *Trem2*, and *Tyrobp*. Particularly, *Csf1r* and *Grn* display the same overexpression pattern, which is, overall, 1/3 of *Trem2* and *Tyrobp* upregulation. Abbreviations: C, cortex; H, hippocampus.

We do not report any significant differential expression in *Arsa*, *Csf1r*, *Eif2b1*, *Eif2b2*, *Eif2b3*, *Eif2b4*, *Eif2b5*, *Htra1*, *Notch3*, and *Trex1* until the development of severe AD pathology, markedly pronounced in the most aggressive AD strain studied, HOTASTPM, homozygous for the Swedish mutation *APP* p.K670N/M671L and *PSEN1* p.M146V, 8 months of age (Fig. 2A–D, Supplementary Tables S3 and S4). Here, *Csf1r* was up to 2 folds significantly overexpressed both in the hippocampus and cortex (log2FC = 1.2 and 1.1; adj *p*-value = 2.5e−07 and 8.7e−05, respectively) and presented a trend at 18 months both in the hippocampus and cortex (log2FC = 0.75 and 0.98; adj *p*-value = 2.7e−04 and 3e−04, respectively), in linear correlation with the most rapid and severe dense-core plaque deposition (0.8 dense-core plaque/month and 0.5 dense-core plaque/month between 4–8 months and 8–18 months of age, respectively) (http://www.mouseac.org/) (Fig. 2A–D, Supplementary Tables S3 and S4). Moreover, *Csf1r* overexpression positively correlated also with tau pathology, suggesting that *Csf1r* upregulation is not Aβ plaque specific. By contrast, *Csf1r* was downregulated when plaque deposition was minimal (HETTASTPM, TAS10, TPM, and TAU, 2m; TAS10 and TAU, 4m) (Supplementary Tables S3 and S4). Importantly, *Csf1r* upregulation relied on microglia infiltration and was coexpressed with other microglia markers such as *Aif1*, *CD68*, *Trem2*, *Tyrobp*, and *Grn*. Particularly, *Csf1r* and *Grn* displayed the same pattern of overexpression, which was between one-third to one-fourth of *Tyrobp* and *Trem2* overall upregulation (Fig. 3, Supplementary Tables S3 and S4).

Raw data are taken from http://www.mouseac.org/

Raw data for this study are taken from http://www.mouseac.org/

### 3.2 Embryonal hippocampi and primary neuronal cortical cultures OGD experiments

We do not report any significant differential expression in the studied genes between APPPS1 and WT embryonal hippocampi and APPPS1 and WT primary cortical neurons after OGD experiments (Supplementary Table S5). This is likely due to the fact that most of the leukodystrophy genes are expressed on microglia, only moderately present in E15 hippocampi and in primary neuronal cortical cultures. In line with this observation, *Csf1r* and its ligands (*Csf1* and *Il34*), *Grn*, *Trem2*, and *Aif2* were significantly overexpressed in both APPPS1 and WT adult hippocampi compared to the embryonal ones (log2FC = 4, 2.45, 7.9, 1.5, 2.24, 3.4 and 4.2, 2.7, 7.9, 1.77, 3.1 and 3.8, respectively) (Supplementary Table S5). By contrast, *Notch3* was up to 2 folds upregulated in both APPPS1 and WT embryonal hippocampi compared to adult hippocampi.

**Supplementary Fig. S2:**
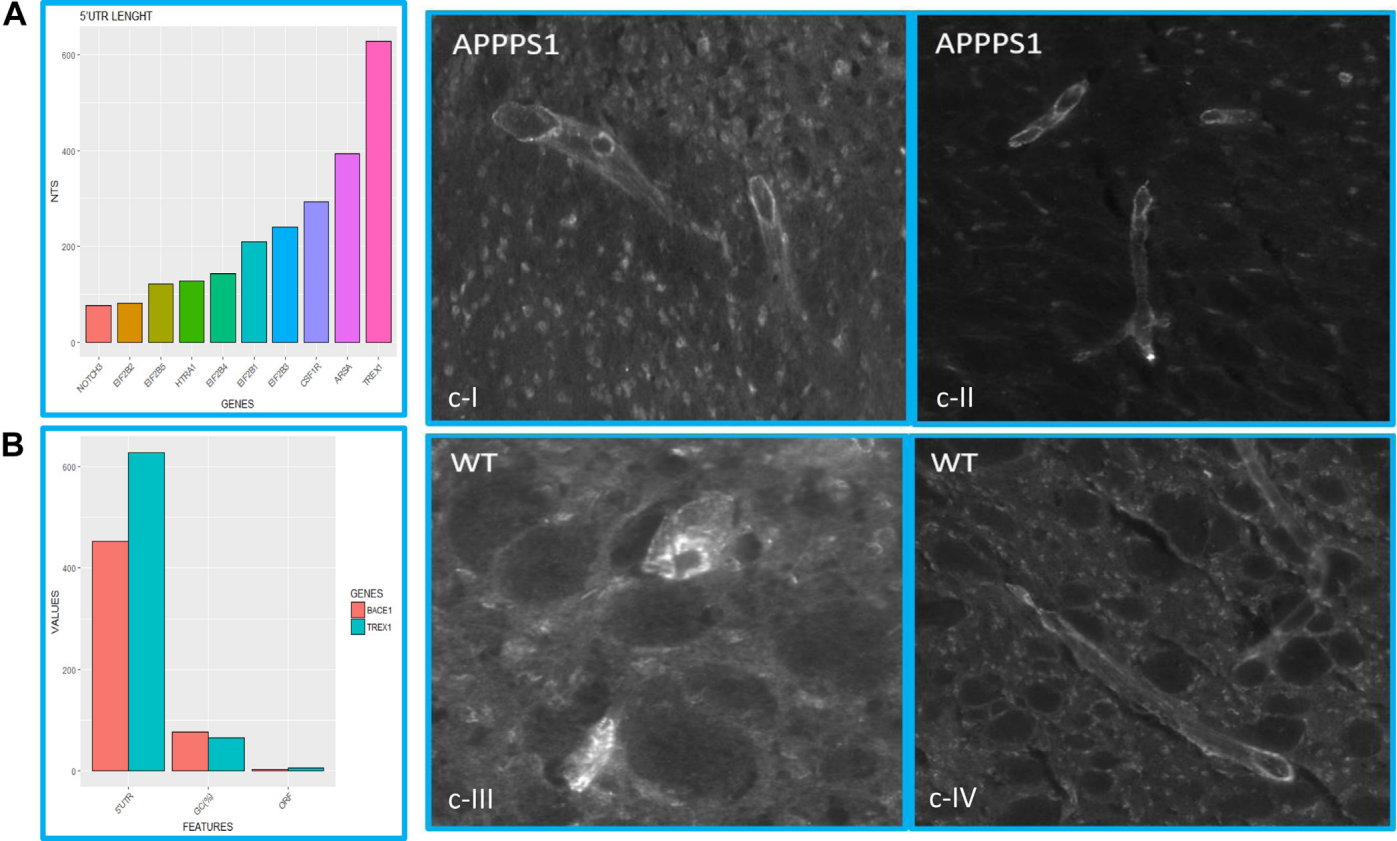
(A) 5′UTR length of the studied adult-onset leukodystrophy genes. *TREX1* presents the longest 5′UTR region (628nts). (B) *TREX1* displays analogous 5′UTR features of *BACE1*, known to be subjected to translational control: (1) 5′UTR length (628 and 453 nts, respectively); (2) GC content (65% and 77%, respectively), and (3) predicted open reading frames (ORFs) (6 and 3, respectively). (CI–IV) Cortical histological sections of APPPS1 and WT mice (aged 2 months), stained with TREX1 antibody (Abnova 68191), which mainly binds to endothelial cells and to a lesser extent neurons. No macroscopical difference has been detected between APPPS1 and WT mice. Abbreviation: WT, wild type.

Moreover, we noticed that *TREX1* 5′UTR displays the typical features of many transcripts, like BACE1, that are translationally controlled by cellular stress (O'Connor et al., 2008): *TREX1* 5′UTR is indeed particularly long (628 nts), GC rich (65%), and predicted to contain 6 upstream open reading frames (http://www.ncbi.nlm.nih.gov/orffinder/) (Supplementary Fig. S2A, B), suggesting *TREX1* transcript might be a target of translation control by one or more stress-activated pathway. Therefore, we have investigated TREX1 protein levels in both APPPS1 and WT adult brains, and we do not report any macroscopically significant difference (Supplementary Fig. S2CI-IV). This may be due to the fact that APPPS1 mice used for these experiments, being 2 months of age, did not display a severe pathology (Supplementary Fig. S1).

### 3.3 Genetic screening

The study population consisted of a total of 332 sporadic and mainly LOAD cases and 676 elderly controls of British and North American ancestry.

We do not report any pathogenic mutation in *APP*, *PSEN1*, and *PSEN2* in our cohort. However, one of the controls was a heterozygous carrier of the protective variant *APP* p.A673T (minor allele frequency [MAF] 7 × 10E−4 in our cohort and MAF 5 × 10E−4 among the European non-Finnish, ExAC database, released on January 13, 2015).

We performed a single-variant and a single-gene association analysis in a predefined set of adult-onset Mendelian leukodystrophy genes (*ARSA*, *CSF1R*, *EIF2B1*, *EIF2B2*, *EIF2B3*, *EIF2B4*, *EIF2B5*, *HTRA1*, *NOTCH3*, and *TREX1).*

A total of 215 single-nucleotide variants have been identified. Among these, 77 (35.8%) were nonsynonymous, 59 (27.4%) were synonymous, and 13 (6%) UTR variants. Among the missense variants, 192 (95%) were very rare (MAF < 1%), 16 (7.9%) were low frequency (1% < MAF < 5%), and 12 (5.9%) were common (MAF > 5%). In addition, we report 4 novel coding variants (*NOTCH3*, p.A2146E, *CSF1R* p.G957R, and p.D565N and *ARSA* p.H425Y). Variant MAF and novel variants were based on ExAC database, European non-Finnish panel, and Exome Variant Server (EVS) European-American panel, released on March 14, 2016, or dbSNP 137 (Supplementary Table S6).

The overall variant frequency in our cohort was in line with the variant frequency reported in the American-European cohort in the Exome Variant server database (Supplementary Table S7).

#### 3.3.1 Single-gene–based analysis

We carried out gene-wide analysis to combine the joint signal from multiple variants (coding variants and flanking UTRs) within a gene and to provide greater statistical power than that for single-marker tests. All the variants (nonsynonymous, synonymous, UTRs, and singletons) located within the studied genes and their exon-intron flanking regions were collapsed together, and their combined effect was studied.

*NOTCH3* is the only significant hit in the c-alpha test (adj *p*-value = 0.01) (Supplementary Table S8a). The signal is driven by a common coding synonymous variant (p.P1521P) of moderate effect size (odds ratio = 1.755, confidence interval = 1.31–2.33), significant after Bonferroni correction (adj *p*-value = 0.02) (Supplementary Table S9). *TREX1* is another hit in the c-alpha test, although nominally significant (adj *p*-value = 0.56), and the signal is mainly driven by a 5′UTR and synonymous (p.Y232Y) variants (Supplementary Tables S8a and S9). None of these variants were predicted to affect the splicing site (http://www.umd.be/HSF/) or a miRNA binding site (http://www.microrna.org/microrna/home.do).

#### 3.3.2 Single-variant–based analysis

A total of 69 rare and low-frequency coding missense mutations were considered in the single-variant–based analysis in the studied genes. Among these, the majority (62.8%) were singletons (Supplementary Table S10).

Moreover, 41 missense variants (59.4%) were described as damaging variants by at least 2 of 3 in silico prediction softwares (SIFT, Polyphen and Mutation Taster).

The study possessed relatively low power to detect a significant association between cases and controls for low-frequency and rare variants, however, we analyzed these variants because we could not preclude the possibility that high-effect risk alleles were present.

*EIF2B4* and *CSF1R* harbor the lowest and highest relative frequency of low-frequency and rare coding variants (mean = 1.27 and 5.13 low-frequency-rare variants per kb of coding sequence, respectively), with 81.25% of the rare and low-frequency coding variability in *CSF1R* clustering in the Ig-like domain (Supplementary Tables S11 and S10).

The main hits, although not significant, are rare variants with moderate to strong effect sizes (0.6 < odds ratio < 2.73) clustering to *EIF2B4*, *NOTCH3*, *TREX1*, and *CSF1R* (Supplementary Table S10).

None of the missense mutations leads to a premature stop codon.

#### 3.3.3 Singletons in *CSF1R* TK domain

We report 2 heterozygous missense mutations in the *CSF1R* TK domain (exons 12–22, aa 582–910) in the discovery cohort (p.L868R and p.E694K), detected in one case and one control, respectively. Moreover, we found 4 likely pathogenic variants in the TK domain flanking regions (aa 538–581 and 911–972): *CSF1R* p.D565N, p.E916K, p.E920D, and p.G957R. We then screened *CSF1R* in an independent follow-up cohort of LOAD and MCI patients, and we identified 2 additional mutations in *CSF1R* TK domain (p.Q691H and p.H703Y) (Table 1).

**Table 1:** Rare variants detected in *CSF1R* TK and TK flanking regions in the discovery and follow-up cohort (a)

Position	rsID	cDNA	Aa change	Domain	ExAc	EVS	AD carriers (tot = 797) (%)	CTRLS carrier (tot = 676)	MT	*p*-value	Adj *p*-value	OR	CI
chr5:149433682	Novel	c.2869G > C	p.G957R	TK flanking region	NA	NA	1 (0.12)	0	Disease_causing	1	1	Inf	0.021–Inf
chr5:149433888	rs34030164	c.2760G > C	p.E920D	TK flanking region	3e-3	2.5e-3	2 (0.25)	7 (1)	polymorphism	0.089	1	0.24	0.024–1.26
chr5:149433902	rs142435467	c.2746G > A	p.E916K	TK flanking region	7e-4	9.3e-4	0	2 (0.29)	Disease_causing	0.21	1	0	0–4.5
chr5:149434851	rs281860278	c.2603T > G	p.L868R	TK	8.9e-5	NA	1 (0.12)	0	Disease_causing	1	1	Inf	0.021–Inf
chr5:149439287	rs111943087	c.2107C > T	p.H703Y^a^	TK	NA	NA	1 (0.12)	0	Polymorphism	1	1	Inf	0.021–Inf
chr5:149439315	rs545858226	c.2080G > A	p.E694K	TK	1.4e-5	NA	0	1 (0.14)	Disease_causing	0.45	1	0	0–33
chr5:149439322	Novel	c.2073G > C	p.Q691H^a^	TK	6e-5	NA	1 (0.12)	0	Polymorphism	1	1	Inf	0.021–Inf
chr5:149441346	Novel	c.1693G > A	p.D565N	TK flanking region	NA	NA	1 (0.12)	0	Disease_causing	1	1	Inf	0.021–Inf

Key: AD, Alzheimer's disease; Aa, amino acid; tot, total; MT, Mutation Taster; Adj *p*-value, adjusted *p*-value, based on Bonferroni correction with 69 rare coding variants; OR, odds ratio; CI, confidence interval. NA, not available; Inf, infinity.

a Variants detected in the follow-up cohort.

*CSF1R* TK mutation carriers (patients E, F, and H) presented a rather homogeneous phenotype (Table 2). All these carriers were LOAD cases displaying memory impairment at the onset. Behavioral and motor signs eventually appeared. In 2/3 patients, cardiovascular problems and strokes preceded the dementia. The neuropathology examination, available for patients H and I, showed aggressive and diffuse neurodegeneration (Braak 6 and CERAD C). Two of 3 carriers were heterozygous for *APOE* ε4 allele and do not report any familial history for dementia. By contrast, patient H was homozygous for *APOE* ε4 allele, had a family history for dementia (4 brothers) and plausibly the combination of these risk factors, likely coupled with a pre-existent cerebrovascular disorder, may explain the earlier age at onset compared to the other patients (64 years). Patient I carried a missense mutation in *CSF1R* TK flanking region (p.G957R) and displayed a different clinical picture, dominated by early-onset dementia (49 years) and language problems at the onset. Despite the small sample size, we do not report any association between age at onset, severity of the disease progression, and disease duration.

**Table 2:** *CSF1R* TK and TK flanking region mutation carrier description

Patient	*CSF1R* mutation	*APOE*	AO-AD	Family history of dementia	Disease duration	First symptom	Behavioral symptom	Motor symptom	Vascular risk factors	Misdiagnosis	CT/MRI	Neuropath
Patient D	p.D565N	34	−92		NA	NA	NA	NA	NA	NA	NA	NA
Patient E	p.Q691H	34	82–89	Negative	7y, rapid deterioration during the last 7 months	Memory problems		No			Symmetric patchy periventricular hyperintensities, mainly pronounced in the frontal lobes	
Patient F	p.H703Y	34	79–82	Negative	3y	Memory problems	Irritability and anxiety	Intermittent mild rigidity, tremor and bradykinesia, mild left hemiparesis	Bilateral severe carotid artery stenosis, vertebrobasilar TIA	Vascular dementia	Hippocampal and temporal lobe atrophies, subcortical microbleeds (right basal ganglia), and small ischemic stroke (left pons), lacunar infarct right parietal lobe, centrum semiovale bilateral lesions	
Patient H	p.L868R	44	64–75	4/4 siblings diagnosed with dementia	11y	Short-term memory problems and dysphasia			Stroke at 65y	Vascular dementia	Severe microbleeding	Extensive Aβ and tau deposition (Braak VI and CERAD C), amyloid angiopathy and focal TDP-43
Patient I	p.G957R	NA	49–57	Negative	8y	Language problem	Aggression and paranoia later in the course of the disease	No		PNFA		Braak VI and CERAD C

Key: Aβ, amyloid beta; AO-AD, age at onset-age at death; symp., symptoms; CT/MRI, computed tomography/magnetic resonance imaging; NA, not available; TIA, transient ischemic attack; PNFA, progressive nonfluent aphasia.

Detailed clinical description was available for 4 patients. The clinical, neuroimaging, and neuropathological features of the carriers are summarized in Table 2.

#### 3.3.4 Patient H (p.L868R)

This male patient deceased at the age of 75 years. He was one of 5 siblings who all survived to old age, of whom 4 experienced memory problems or received a diagnosis of dementia or AD. The informant reported he experienced sudden decline following a stroke at 65 years. He had obvious short-term memory problems and dysphasia. At this stage, he was considered to have probable vascular dementia. Pathological examination of the brain concluded this patient had a high probability of AD. Neurofibrillary tangle stage was consistent with Braak stage VI, while plaque pathology met CERAD criteria for score C. In addition, there was evidence of amyloid angiopathy, focal TDP-43 positivity, and occasional glial inclusions.

#### 3.3.5 Patient E (p.Q691H)

**Fig. 4:**
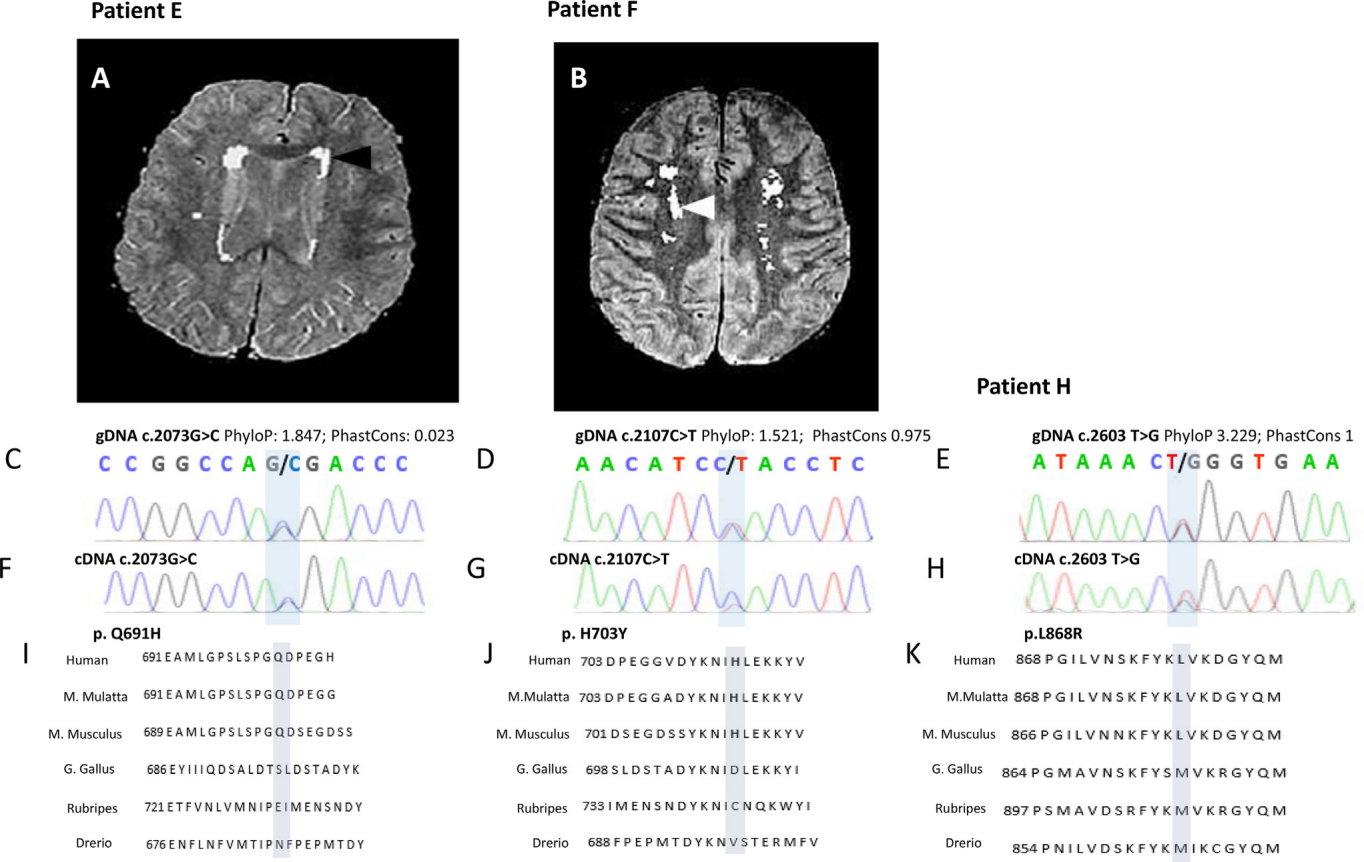
MRI scans, Sanger sequencing validation, and mutation domain conservation for patients E, F, and H. (A–B) Coronal T2-weighted MRI scan of patients E and F (for both taken 2y after onset of symptoms, aged 84y and 81y, respectively) showing symmetric patchy periventricular hyperintensities, mainly pronounced in the frontal lobes (A) and bilateral lesions localized to the centrum semiovale (B) (arrows). (C–E) Genomic DNA Sanger sequencing validation of *CSF1R* c.2073G>C, c.2107C>T, and c.2603 T>G mutations. (F–H) cDNA Sanger sequencing validation of c.2073G>C, c.2107C>T, and c.2603 T>G mutations. For patients F and H, cDNA sequence highlights a possible allelic imbalance, supporting the likely functional effect of gDNA c.2107C>T and gDNA c.2603 T>G mutations. (I–K) Conservation of p.Q691H, p. H703Y, and p.L868R in different species. PhastCons and PhyloP scores range between 0–1 and −14 to +6, respectively. For PhastCons, the closer to one, the more conserved; for PhyloP, conserved sites are assigned positive scores. Abbreviation: MRI, magnetic resonance imaging.

This patient deceased at the age of 89 years. She complained of memory problems at the age of 82 years, and 2 years later underwent an MRI scan, which showed symmetric patchy periventricular hyperintensities, mainly pronounced in the frontal lobe (Fig. 4A). Following annual visits involving neuropsychiatric testing, she received a diagnosis of AD at the age of 86 years. In the 3 years following her diagnosis, her symptoms were quite stable. She experienced a rapid deterioration in the last 7–8 months before her death.

#### 3.3.6 Patient F (p.H703Y)

This patient had a strong history of cardiovascular disease and reported memory symptoms, at the age of 79 years, followed by irritability and anxiety and 2 years later received a clinical diagnosis of probable AD. Computed tomography of the brain showed supratentorial atrophy, temporal lobe atrophy, and slight vascular changes. The patient also experienced intermittent motor symptoms that included mild rigidity, tremor, and slowness of movement. The MRI scans showed central and cortical atrophies and mild to moderate medial temporal lobe atrophy, as well as a small old hemorrhage, ischemic lesions, and bilateral lesions localized to the centrum semiovale (Fig. 4B).

Patient I's detailed description is in the Supplementary data.

### 3.4 Tissue expression of CSF1R

**Fig. 5:**
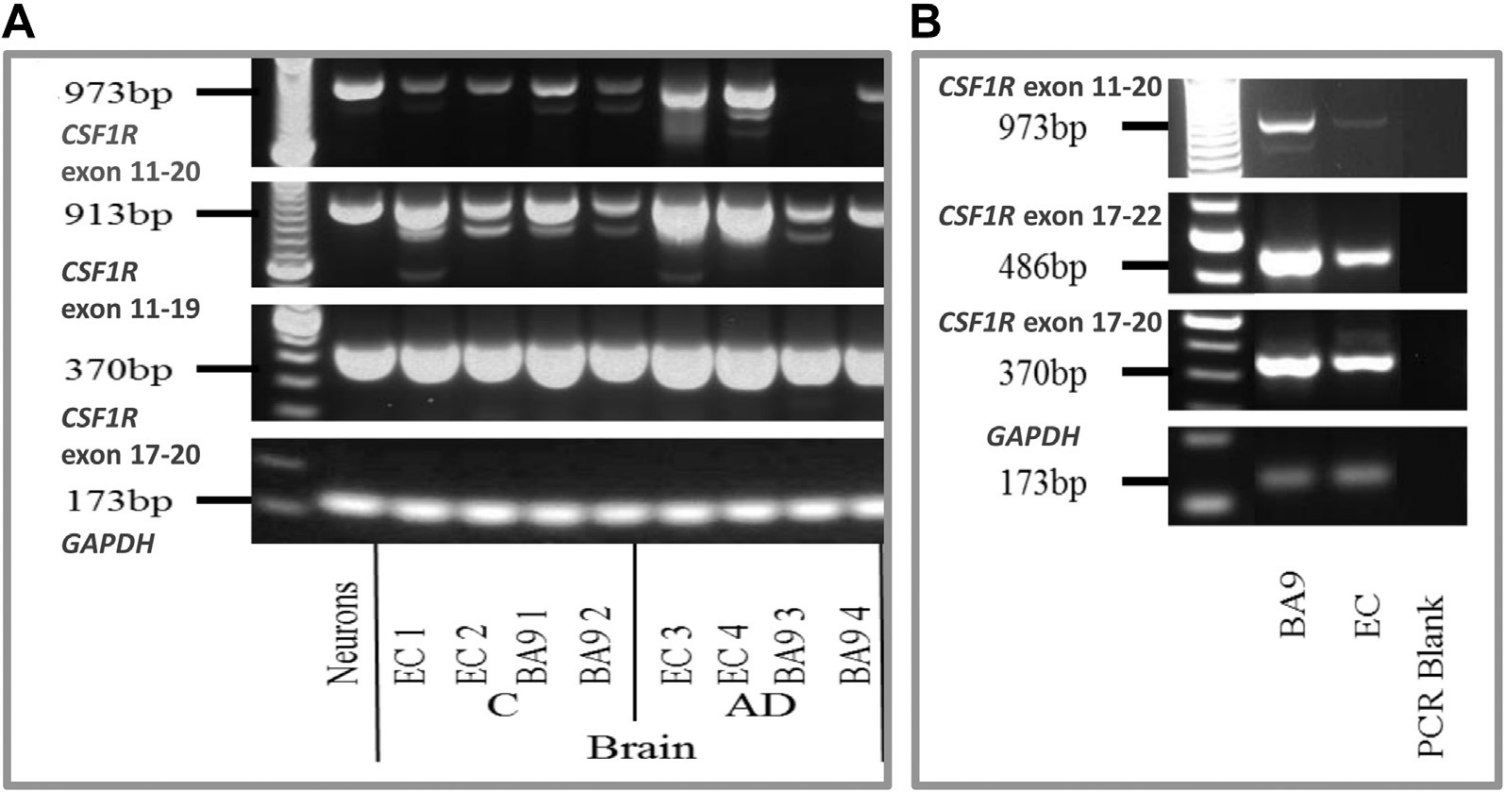
Tissue expression of *CSF1R*. (A) Relative *CSF1R* and *GAPDH* expression assessed by RT-PCR in postmortem brain RNA from normal control and AD individuals. (B) Expression of *CSF1R* transcripts and the house-keeping gene *GAPDH* in the EC and BA9 preassociation cortex (BA9) from patient H (c.2603T>G, p.L868R). RT-PCR for *CSF1R* exon 11 to 20, exon 11 to 19, exon 17 to 20, and exon 17 to 22 as indicated. Abbreviations: EC, entorhinal cortex; BA, Brodmann area; AD, Alzheimer’s disease; C, normal control; RT-PCR, Real Time PCR.

We followed up our findings checking *CSF1R* expression in LOAD and control brain samples. We selected the entorhinal cortex (EC) and BA9 preassociation cortex (BA9) because the brain regions primarily affected by AD spreading pathology (Khan et al., 2014). *CSF1R* was overexpressed in AD EC compared to AD BA9 preassociation cortex and control brains (Fig. 5A).

It was not possible to quantitatively compare levels of *CSF1R* in all the 3 *CSF1R* TK mutation carriers due to a lack of available brain tissue. However, cDNA Sanger Sequencing revealed a possible allelic imbalance, with the WT allele normally expressed and the mutated one only moderately both for patient F (p.H703Y) and patient H (p.L868R), suggesting a functional role of these mutations (Fig. 4G–H). RNA from the EC and the BA9 preassociation cortex was available for patient H and showed significantly lower expression of *CSF1R* (1) in the EC compared to BA9 preassociation cortex and (2) in patient H's EC compared to other AD patients and controls for all *CSF1R* primers tested (Fig. 5A–B).

## 4 Discussion

Mendelian adult-onset leukodystrophies clinically resemble common dementias such as AD, potentially implying they may be influenced by shared genetic risk factors.

To comprehensively investigate this hypothesis, we applied a combination of gene expression analysis in different AD mouse strains at diverse developmental stages (http://mouseac.org/) and single-variant–based and single-gene–based genetic screening in a cohort composed of 332 LOAD cases and 676 elderly controls from the United Kingdom and United States (Fig. 1). Divergent gene expression between AD and WT mouse strains was only detected in aged mice with severe dense-core plaque deposition (Fig. 2A–D, Supplementary Tables S3 and S4).

*Csf1r* was the only gene displaying a significant differential expression between AD and WT mouse strains. It was up to 2 folds significantly overexpressed both in the hippocampus and cortex in HOTASTPM mice aged 8 months (log2FC = 1.2 and 1.1; adj *p*-value = 2.5E−07 and 8.7E−05, respectively), and its overexpression linearly correlated with the rapidity of dense-core plaque deposition rather than with their overall load (Fig. 2A–D, Supplementary Tables S3 and S4). By contrast, *Csf1r* was downregulated when the pathology was minimal or absent (Supplementary Tables S3 and S4), suggesting that *Csf1r* upregulation was tightly driven by and consequential to dense-core plaque formation and, to a lesser extent, neurofibrillary tau tangles.

Importantly, *Csf1r* expression pattern relied on microglia infiltration as overexpression of *Aif1* suggested (Fig. 2A–B, Supplementary Tables S3 and S4). *Csf1r* was coexpressed and shared the same expression pattern with *Trem2*, *Tyrobp*, and *Grn*, critical genes expressed on microglia, whose loss of function mutation in homozygosity is causative for adult-onset leukodystrophies such as polycystic lipomembranous osteodysplasia with sclerosing leukoencephalopathy (PLOSL) (*TREM2* and *TYROBP*) (Paloneva et al., 1993), and in heterozygosity causes frontotemporal lobar degeneration with TDP-43 inclusions (FTLD-TDP43) (*GRN*) (Baker et al., 2006) or is a significant risk factor for sporadic AD (*TREM2*) (Guerreiro et al., 2013), and whose overexpression is protective and limits AD neuropathology through a very effective clearance of Aβ plaques (*GRN* and *TREM2*) (Minami et al., 2014). Notably, in all the strains, *Csf1r* and *Grn* degree of expression correlated, and this was generally one-third of *Tyrobp* and *Trem2* overall upregulation (Fig. 3, Supplementary Tables S3 and S4). This effect was not simply due to the aging process: we do not report significant differential expression in *Csf1r*, *Grn*, *Trem2*, and *Tyrobp* in the cortex and hippocampus of WT mice between 2 and 18 months of age (−0.04 < log2FC < 0.2 and 0.04 < log2FC < 0.4 in the hippocampus and cortex, respectively) (Supplementary Tables S3 and S4). Importantly, *CSF1R*, *TREM2*, and *TYROBP* have been already shown to cointeract (Otero et al., 2009) (https://string-db.org/). Here, we report *GRN* as an additional potential key player and its coexpression on microglia strengthens its synergic function. Therefore, this may imply that *CSF1R*, in concert with *TREM2*, *TYROBP*, and *GRN*, plays a key role in Aβ plaque removal, hypothesis supported by previous literature, reporting *CSF1R* overexpressed in AD patients particularly around senile plaques and taking part in Aβ removal (Akiyama et al., 1994) (Murphy et al., 2000) (Mizuno et al., 2011) (Boissonneault et al., 2009).

By contrast, no differential expression of any adult-onset leukodystrophy gene was observed in the E15 hippocampi and primary cortical neurons after OGD experiments between APPPS1 and WT strains, likely given the fact that these genes, although expressed in neurons, mainly exert a critical role on microglia (*TREX1* and *CSF1R*), astrocytes (*NOTCH3* and *HTRA1*), and endothelial cells (*NOTCH3, HTRA1, TREX1, EIF2B2, EIF2B3,* and *EIF2B5*) (http://web.stanford.edu/group/barres_lab/brain_rnaseq.html) that are minimally present in E15 hippocampi and primary cortical neuronal cultures.

In our discovery and validation cohorts, we detected 3 rare coding variants in the TK domain of *CSF1R* in 3 LOAD cases, one of these neuropathologically confirmed. Moreover, we report 2 cases harboring rare mutations in the TK flanking regions (aa 538–580 and aa 911–972, encoded by exon 12 and 22, respectively), where an additional causative mutation for HDLS has been recently described (c.1736G>A, p.R579Q) (Ghadiri et al., 2014). These variants are very likely pathogenic: they cluster to highly conserved domains among different species (average PhyloP and PhastCons scores = 2.2 and 0.6, respectively) (Fig. 4I–K) and have been detected only in cases.

Moreover, *CSF1R* p.L868R is a functional mutation as it was associated with a reduced expression of the mutated allele (Fig. 4H) and decreased *CSF1R* expression in the EC, a region primarily affected in AD and generally displaying *CSF1R* upregulation in AD patients (Fig. 5A and B). Importantly, a different amino acid change, clustering within the same codon (p.L868P) has been reported as *de novo* mutation, causative for HDLS (Rademakers et al., 2011). Remarkably, to date, any missense mutation in Mendelian gene domains harboring heterozygous causative mutations for autosomal dominant disorders such as familial AD and FTD (*APP*, *PSEN1*, *PSEN2*, and *MAPT*) has always been reported as pathogenic (Supplementary Table S12, http://www.molgen.ua.ac.be/ADMutations/). The only exception is represented by *APP* p.A673T, which is a very rare protective factor for AD (Jonsson et al., 2012). In addition, an intronic single-nucleotide polymorphism (SNP) in *CSF1R*, rs1010101, displayed a trend toward association (adj *p*-value = 2E−4), in a genome-wide association study performed in Caucasian LOAD patients (Wijsman et al., 2011), supporting *CSF1R* possible role in LOAD progression.

*CSF1R* mutation carriers presented a homogeneous phenotype, closely resembling HDLS. First, the symptom at onset was a memory deterioration followed by behavioral changes in 3/3 carriers. Second, T2-weighted MRI, available for 2 patients, showed symmetric patchy periventricular hyperintensities, mainly pronounced in the frontal lobe (patient E) and bilateral lesions localized to the centrum semiovale (patient F) (Fig. 4B), that represent common MRI findings in HDLS patients (Ghadiri et al., 2014), (Rademakers et al., 2011) (Boissé et al., 2010) (Mateen et al., 2010). Finally, senile plaques, amyloid angiopathy, and tau tangles have been reported also in the cortex and hippocampus of 2 familial and 1 sporadic HDLS patients (Baba et al., 2006) (Browne et al., 2003). Importantly, these 3 HDLS patients displaying AD neuropathological hallmarks presented an overall early age at onset (average 54 years [range 78–32 years]), developed Parkinsonism, atypical Parkinsonism, and motor impairment with increasing rigidity (Supplementary Table S13). By contrast, only patient F (20%) displayed intermittent mild rigidity, tremor, and bradykinesia, arguing for Parkinsonism, however within a neurological picture already dominated by cerebrovascular disorders, and patient H reported no sign of motor impairment besides 2 falls in few months. Nevertheless, although an earlier age at onset, Parkinsonism and distinctive motor features may be more common in HDLS patients presenting AD neuropathology than AD cases carrying *CSF1R* TK mutations; the average disease duration for both these HDLS and AD patients was 7 years. Therefore, in the absence of accurate differential diagnostic criteria, this combination of clinical, neuroimaging, and neuropathological features strikingly overlapping makes the definitive neurological diagnosis a real conundrum. The fact that potentially pathogenic mutations in the TK domain in heterozygosity may be detected either in databases and apparently healthy controls, may give rise to HDLS or may be rare risk factors for AD may be due to different factors modifying the mutation penetrance (Karle et al., 2013). Analogously to *GRN* missense mutations in AD and FTD, *CSF1R* mutation penetrance may be influenced by *APOE* genotype, aging, disease duration, or comorbidities such as cerebrovascular accidents, for which *CSF1R* has been already shown to play a critical protective role (Luo et al., 2013). In addition, it may be plausible that most HDLS patients may not display AD neuropathology due to the rapid progression of the disease (Baba et al., 2006).

Finally, *NOTCH3* was a significant hit in the gene-based analysis (c-alpha test, adj *p*-value = 0.01). The signal was driven both by a common synonymous variant (p.P1521P) (Supplementary Table S9) that may influence gene expression (Sauna and Kimchi-Sarfaty, 2011) and 3 rare coding variants with large effect size (p.V1952M, p.V1183M, p.H170R, 2.73 <0R < 1.63), whose carrier frequency was between 2 and 3 times higher in cases compared to controls, although not significant (Supplementary Tables S6 and S10). Importantly, these rare variants (p.V1952M, p.V1183M, and p.H170R) have been already reported to be significantly associated with severity of white matter lesions in elderly with hypertension (Schmidt et al., 2011), suggesting a potential role as disease modifier in LOAD. In addition, we report a heterozygous pathogenic gain of cysteine mutation in *NOTCH3* (p.R578C) detected in 1 control and already reported in a Korean patient with clinical suspicious CADASIL, implying that the penetrance of *NOTCH3* mutations is variable (Kim et al., 2014).

Therefore, although canonical *NOTCH3* mutations causative for CADASIL are highly stereotyped: (1) cluster in epidermal growth factor–like repeat domains, (2) in exons 3 and 4, and (3) consist in the gain or loss of cysteine; nevertheless, our study reports a possible synergetic effect of common and rare variants in *NOTCH3* potentially influencing AD susceptibility through an increased risk for small vessel disease or white matter lesions. Our hypotheses are supported by a growing body of evidence showing that (1) *NOTCH3* common variants (rs1043994, rs10404382, rs10423702, and rs1043997) are significantly associated with white matter lesions in elderly with hypertension (Schmidt et al., 2011) and (2) rare noncysteine mutations may be pathogenic as they have been reported in Korean and Japanese CADASIL patients, in a French case with small vessel disease, and have been associated to severe white matter lesions in elderly patients (Fouillade et al., 2008, Mizuno et al., 2008, Schmidt et al., 2011). Importantly, our findings add evidence to the pathogenic link between AD and CADASIL, displaying clinical shared features and rarely, as only few cases have been reported, neuropathological hallmarks characterized by Aβ plaques, amyloid angiopathy, and neurofibrillary tangles (Guerreiro et al., 2012, Gray et al., 1994, Paquet et al., 2010, Thijs et al., 2003). Biologically, presenilins cleaving both APP and NOTCH3 may bridge the gap between AD and CADASIL. However, whether *NOTCH3* mutations or differential expression may accelerate a pre-existing AD or AD may contribute to CADASIL exacerbation remains to be elucidated.

In summary, adult-onset Mendelian leukodystrophy genes are not common factors in AD, therefore the genetic screening plays a pivotal role in the differential diagnosis. However, genetically diagnosed HDLS and CADASIL patients may display clinical, neuroimaging, and neuropathological features meeting the diagnostic criteria for AD, leaving the definitive diagnosis a significant challenge. Here, we report neuropathologically confirmed AD patients carrying likely pathogenic mutations in *CSF1R* TK domain and a potential association between AD and *NOTCH3*. Our study provides compelling evidence that HDLS, CADASIL, and AD may represent shades of the same disease spectrum. Moreover, we support previous studies, suggesting that *CSF1R*, in concert with *TREM2*, *TYROBP*, and *GRN*, may play a critical role in Aβ plaque clearance and therefore may represent a pivotal, although rare, genetic factor influencing AD susceptibility. Given the very rare frequency of *CSF1R* TK pathogenic mutations detected in the screened patients (0.3% LOAD carriers), our hypotheses should foster genetic screening in larger cohorts of both early-onset AD and LOAD cases and functional studies.

## Disclosure statement

All the authors declare no competing financial or personal interests that can influence the presented work. However, MAN's participation is supported by a consulting contract between Data Tecnica International and the National Institute on Aging NIH, Bethesda, MD, USA, as a possible conflict of interest, and he also consults Illumina Inc, the Michael J. Fox Foundation, and the University of California Healthcare among others.
