# Supplementary material for: Mendelian adult-onset leukodystrophy genes in Alzheimer's disease: critical influence of CSF1R and NOTCH3
Source: Neurobiol Aging. 2018 Jun;66:179.e17–29. doi: 10.1016/j.neurobiolaging.2018.01.015 (PMC5937905; doi:10.1016/j.neurobiolaging.2018.01.015)
Supplement: Supplementary Materials and Methods [file mmc1.docx]

**Supplementary**

**MATERIAL AND METHODS**

**Isolation of Mouse Cortical Neurons and Neuronal Cell Culture**

Primary cortical neurons were derived from C57BL6/N and APPPS1 embryos (E15) and cultured using Neurobasal medium with B27 supplement as described (Harms et al., 2007)

**Oxygen–Glucose Deprivation**

Neuronal cultures were subjected to combined oxygen and glucose deprivation as described previously (Harms et al., 2007). In brief, cultures were washed twice and placed in an OGD chamber ‘IN VIVO_2_ 300' (Ruskinn, Pencoed, UK) with 5% CO_2_/0.3% O_2_ with a buffer free of glucose for 3 hours.

**Mouse Region Isolation**

Four APPPS1 mice and 4 wild type (WT) mice (C57BL6/N) (2 months of age) were sacrificed with cervical dislocation, followed immediately by post-mortem dissection of hippocampus from one hemisphere. The other hemisphere was preserved for immunohistochemistry. Twenty embryonal (E15) hippocampi were isolated and pooled together both for APPPS1 and WT embryos. The dissected tissues were immersed in RNA later (Invitrogen) and stored at -80 °C for later use for RNA extraction. Total RNA was extracted using miRNeasy Kit (Qiagen, Cat # 217004).

All experiments were repeated twice independently, and the data were presented as the means ± SD. Statistical comparisons were conducted using paired Student’s t test and false discovery rate (FDR) correction was applied. P-value < 0.05 and ǀlog2FCǀ≥1 were considered statistically significant

**Sanger sequencing**

Mutations in *CSF1R* TK domain and flanking regions were validated with Sanger Sequencing*. CSF1R* was screened in an additional follow-up cohort composed of 465 AD and MCI cases. Exonic primers were designed to screen the *CSF1R* coding region (Exons 12-21). The target region encompassed the mutations previously been found to cluster in *CSF1R* and the key tyrosine trans-autophosphorylation residues in the cytoplasmic domain (5’- ***CACGACGTTGTAAAACGAC***CAGCTGCCTTACAACGAGAA

-3’ and 5’- ***GGATAACAATTTCACACAGG***AGGCCTGCATGATGCTGTAT -3’; 973bp product; ***M13 sequencing tags***). A QuantiFast SYBR Green PCR protocol (Qiagen) was carried out using the following conditions: 2μl 1:5 diluted cDNA was included in 20μl reactions containing 1XQuantiFast SYBR Green PCR master mix; 1mM forward and reverse primers and thermocycling at 95°C for 5 mins, followed by 42 cycles of 95°C for 10 secs, 70°C for 30 secs and 60°C for 30 secs. PCR reactions were purified by paramagnetic bead-based SPRI® and subjected to Sanger sequencing using M13 primers and Big Dye® capillary terminator chemistry sequencing (Applied Biosystems) according to standard protocols (Beckman Coulter Genomics).

**Bioinformatics**

Sequence alignment and variant calling were performed against the reference human genome (UCSC hg19). Alignment was performed with the use of CASAVA software and variant calling was performed with the use of SAMtools (Li et al., 2009) and GATK (McKenna et al., 2010). Paired end sequence reads (2x100bp paired end read cycles) were aligned using the Burrows-Wheeler aligner (Li and Durbin, 2009). Format conversion and indexing were performed with Picard ([www.picard.sourceforge.net/index.shtml](http://www.picard.sourceforge.net/index.shtml)). GATK was used to recalibrate base quality scores, perform local re-alignments around indels and to call and filter the variants (McKenna et al., 2010). VCFtools was used to annotate gene information for the remaining novel variants. We used ANNOVAR software to annotate the variants (Wang et al., 2010). Variants were checked against established databases (1000 Genomes Project and dbSNP v.134). The protein coding effects of variants was predicted using SIFT, Polyphen2 and SeattleSeq Annotation (gvs.gs.washington.edu/SeattleSeqAnnotation). All variants within the coding regions of the 10 candidate genes (*ARSA* [NM_000487]; *CSF1R* [NM_005211]; *EIF2B1* [NM_001414]; *EIF2B2* [NM_014239]; *EIF2B3* [NM_001261418]; *EIF2B4* [NM_001034116]; *EIF2B5* [NM_003907]; *HTRA1* [NM_002775]; *NOTCH3* [NM_000435] and *TREX1* [NM_016381] have been collected and analysed. Indels were excluded from the merged dataset because not targeted in the ADNI subcohort (**Figure 1**).

**Statistical Analysis**

In the single-variant analysis, allele frequencies were calculated for each low frequency and rare coding variants in cases and controls and Fisher’s exact test on allelic association was performed. To study the joint effect of the variants detected, we performed a gene-based analysis with SKAT and c-alpha tests and we analyzed together for each gene the whole spectrum of allelic variability (common, low frequency, rare, coding and non-coding). Low frequency and rare variants were defined as having a 1%<MAF<5% and MAF<1%, respectively, either in cases or controls. All computations, C-alpha and SKAT tests were performed in R (version x64 3.0.2, <http://www.r-project.org/>) and PLINK/SEQ.

A p-value of 0.05 was set as a nominal significance threshold. Based on multiple testing correction, the thresholds for single variant and gene-based association tests are defined by p-value=7.24E-4 (0.05/69 [total number of coding low frequency and rare variants detected in our study]) and 5xE-3 (0.05/10 genes), respectively.

MouseAC data have been analyzed and FDR correction was applied.

**RNA and DNA extraction for human samples**

After thawing PAXgeneTM tubes at room temperature, total RNA from blood was extracted using the PAXgeneTM blood RNA kit (Qiagen) and from brain tissue using the miRNeasy Mini Kit (Qiagen), according to the manufacturer’s protocols. The quality of the RNA was determined using the 2100 Bioanalyser (Agilent Technologies) and the concentration determined by NanoDropTM 1000 spectrophotometer (NanoDrop Technologies). Blood samples with a RNA integrity number (RIN) 7.0 were used in subsequent analyses, which in some cases meant re-extracting from a duplicate blood sample taken at the same visit or from blood collected at subsequent visits. cDNA was made using 250ng RNA with the QuantiTect Reverse Transcription kit (Qiagen) according to the manufacturers protocol.

**RNA Isolation for mouse samples and Real-Time PCR**

Total RNA from the embryonal hippocampus (E15) and adult (2months of age) APPPS1 and WT hippocampus was isolated using RNeasy Mini kit (QIAGEN). The quality and the concentration of the total RNA was determined using a Nanodrop Spectrometer (A260:A280 and A260:A230 ratios). For real-time PCR analysis, 750ng total RNA from each sample was used for first-strand cDNA synthesis using SuperScript III (Invitrogen). cDNA from each sample was amplified via real-time PCR and normalized against Actin, using LightCycler 480 Instrument II (Roche). Sequence of primers utilized is listed in **Table S14**. mRNA levels for each experimental group were quantified using the comparative CT method.

**RESULTS**

**Patient I (p.G957R)**

This male patient died aged 57 years. No remarkable family history was noted. The onset of problems occurred at 49 years with speech production impairment which progressed over the next few years with subsequent “word finding difficulty” and difficulty with comprehension of speech in specific contexts. No behavioural symptoms were noted at this time and memory/other cognitive domains were relatively intact. The patient was diagnosed with progressive non-fluent aphasia (PNFA) subtype of frontotemporal lobar degeneration (FTLD). The patient presented with hesitant speech with phonemic errors and agrammatism although intact polysyllabic word repetition. On bedside testing, a deficit in working memory but intact episodic memory, limb and orofacial praxis and visuospatial function were noted with MMSE 27/30. Neuropsychology tests showed non-fluent aphasia with phonemic errors, but verbal/visual memory, naming, calculation, executive function all relatively intact. EEG showed left fronto-temporal slow activity with intact alpha rhythm. MRI scans showed generalized volume loss but no regional atrophy and later loss but asymmetrical widening of right frontal perisylvian and superior temporal sulci.

Follow-up showed progressive speech production impairment with worsening writing (agrammatic and phonemic errors). Impaired repetition of polysyllabic words. The patient developed difficulties with planning, executing complex tasks and managing time together with possible personality changes.

The post-mortem examination revealed diffuse Aβ plaques and neurofibrillary tangles, corresponding to Braak and Braak stage VI and CERAD C. NIA-Reagan criteria provided a high likelihood that dementia was due to Alzheimer’s disease.

References

Harms, C., Albrecht, K., Harms, U., Seidel, K., Hauck, L., Baldinger, T., Hübner, D., Kronenberg, G., An, J., Ruscher, K., Meisel, A., Dirnagl, U., von Harsdorf, R., Endres, M., Hörtnagl, H., 2007. Phosphatidylinositol 3-Akt-kinase-dependent phosphorylation of p21(Waf1/Cip1) as a novel mechanism of neuroprotection by glucocorticoids. J. Neurosci. 27, 4562–4571. https://doi.org/10.1523/JNEUROSCI.5110-06.2007

Li, H., Durbin, R., 2009. Fast and accurate short read alignment with Burrows-Wheeler transform. Bioinformatics 25, 1754–1760. https://doi.org/10.1093/bioinformatics/btp324

Li, H., Handsaker, B., Wysoker, A., Fennell, T., Ruan, J., Homer, N., Marth, G., Abecasis, G., Durbin, R., 1000 Genome Project Data Processing Subgroup, 2009. The Sequence Alignment/Map format and SAMtools. Bioinformatics 25, 2078–2079. https://doi.org/10.1093/bioinformatics/btp352

McKenna, A., Hanna, M., Banks, E., Sivachenko, A., Cibulskis, K., Kernytsky, A., Garimella, K., Altshuler, D., Gabriel, S., Daly, M., DePristo, M.A., 2010. The Genome Analysis Toolkit: a MapReduce framework for analyzing next-generation DNA sequencing data. Genome Res. 20, 1297–1303. https://doi.org/10.1101/gr.107524.110

Wang, K., Li, M., Hakonarson, H., 2010. ANNOVAR: functional annotation of genetic variants from high-throughput sequencing data. Nucleic Acids Res. 38, e164. https://doi.org/10.1093/nar/gkq603
