# Supplementary material for: Mendelian adult-onset leukodystrophy genes in Alzheimer's disease: critical influence of CSF1R and NOTCH3
Source: Neurobiol Aging. 2018 Jun;66:179.e17–29. doi: 10.1016/j.neurobiolaging.2018.01.015 (PMC5937905; doi:10.1016/j.neurobiolaging.2018.01.015)
Supplement: Supplementary Tables [file mmc2.docx]

| **DISEASE** | **GENE** | **PREVALENCE** | **MUTATION** | **INH** | **ONSET** | **FIRST SIMPTOM** | **DISEASE DURATION** | **ADDITIONAL NEURO. SYMPT.** | **MISDIAGNOSED**  **WITH** | **MRI findings** | **Neuropath** |
| --- | --- | --- | --- | --- | --- | --- | --- | --- | --- | --- | --- |
| CADASIL | *NOTCH3* | 2 and 5 in 100,000 | gain or loss of a cysteine | AD | adult onset (third decade) | acute unilateral visual loss, early-onset strokes in 43% of patients | 30 years | recurrent subcortical infarcts (strokes) , migraine (40% of patients) , seizures (2-10% of patients) , gait abnormalities, psychiatric | AD, HDLS | lacunar infarctions, microbleeds and subcortical white matter changes. No involvement of the anterior temporal lobes | lacunar infarct-like lesions, extensive demyelination and widespread hyalinization of arteriolar walls with karyolysis and granular deposits |
| CRV | *TREX1* | <1/1,000,000 | missense, LoF | AD | adulthood | decreased visual acuity, retinal vasculopathy | death occurs 5 to 10 years after onset | stroke , migraine , seizures, leg hyperreflexia, hemiparesis, psychiatric disturbances | AD | periventricular and subcortical hyperintensities | periventricular white matter lesions, subcortical lesions with edema , pseudotumors |
| CARASIL/ CADASIL2 | *HTRA1* | 50 people reported, primarily in Japan and China | missense, LoF | AR/AD | range 14 to 44 years | subcortical ischemic events and cognitive decline | rapidly progressive course | pyramidal signs (spasticity, hyperreflexia ), extrapyramidal signs (rigidity) | AD | early confluent or confluent diffuse white matter hyperintensities sometimes associated with multiple lacunar infarcts and microbleeds. Anterior temporal regions and U fibers are spared | demyelination of the cerebral white matter with some preservation of U fibers and small cystic foci in the white matter |
| HDLS | *CSF1R* | <1 / 1,000,000 | missense,LoF | AD | generally 40-60y, but also sporadic cases with age-at onset above 65y | rapidly progressive cognitive decline | death within 6 years after onset | pyramidal signs (spasticity, hyperreflexia ), extrapyramidal signs (rigidity, bradykinesia), postural instability, shuffling gait, mutism, apraxia | FTD, AD, MS, CADASIL; PD, Pick disease | deep white matter lesions predominantly bilateral, but asymmetric in the frontal/parietal regions. Cortical atrophy and involvement of the corpus callosum, corticospinal tracts may be involved late in the disease course. | frontotemporal cortical atrophy, ventriculomegaly, neuronal loss, and hypertrophic astrogliosis in the superficial and deep white matter, loss of axons, dystrophic axons, and axonal spheroids containing neurofilaments. |
| MLD | *ARSA* | 1/40, 000-100, 000 | missense | AR | after puberty | schizophrenia–like symptoms | 12 years | loss of speech, dysarthria, hyporeflexia (early stages), dystonia , chorea , ataxia , spastic tetraplegia (late stages) , seizures, progressive | schizophrenia, AD | subcortical brain atrophy and periventricular white matter changes |  |
| VWM disease | *EIF2B1*, 2, 3, 4, 5 | unknown | homozygous-compound heterozygous missense mut | AR | onset may also occur in early infancy, adolescence, or adulthood | psychiatric symptoms | unknown | seizures , spasticity | schizophrenia, AD | diffuse hyperintense lesions in the cerebral white matter, most prominent in the frontal lobe | cystic degeneration of the white matter with preserved cortex |

**Table S1. Clinical, neuroimaging and neuropathological synopsis of the 10 adult-onset leukodystrophies studied. Most of the information is taken from** [**https://omim.org/**](https://omim.org/)**. Inh, inheritance; LoF, loss of function; FTD, frontotemporal dementia; AD, Alzheimer’s disease; MS, multiple sclerosis; PD, Parkinson’s disease**

| ***CSF1R* Follow-up cohort** | **MCI** | **AD** |
| --- | --- | --- |
| Number of Samples sequenced | 169 | 296 |
| Gender (F (%)/M (%)) | 91(53.8)/77(45.5) | 178(60.13)/118(39.8) |
| Mean age at baseline in years (±SD) | 75.6 (6.2) | 76.7 (6.9) |
| Mean disease duration in years (±SD) | NA | 3.3 (2.6) |
| Mean baseline MMSE (±SD) | 27.4 (1.8) | 21.7 (4.9) |
| Mean baseline ADAS-Cog (±SD) | NA | 19.1 (12.4) |
| Mean baseline CDR sum of boxes (±SD) | 1.3 (0.8) | 5.9 (3.0) |

**Table S2**. Participants in the AddNeuroMed and the King’s Health Partners/Maudsley BRC and Dementia Case Registry at King’s Health Partners. MCI, mild cognitive impairment; AD, Alzheimer’s disease; F, female; M, male; SD, standard deviation.

| **gene** | **N. variants in the discovery cohort (tot = 1008)** | **Frequency in the discovery cohort (%)** | **N. variants in EVS**  **(tot = 4300 European-American)** | **Frequency in EVS European-American cohort (%)** |
| --- | --- | --- | --- | --- |
| *HTRA1* | 8 | 0.79 | 55 | 1.27 |
| *TREX1* | 11 | 1.09 | 35 | 0.81 |
| *EIF2B4* | 11 | 1.09 | 69 | 1.6 |
| *EIF2B3* | 12 | 1.19 | 57 | 1.32 |
| *EIF2B2* | 13 | 1.29 | 35 | 0.81 |
| *EIF2B5* | 18 | 1.78 | 87 | 2.0 |
| *EIF2B1* | 20 | 1.98 | 45 | 1.04 |
| *ARSA* | 29 | 2.87 | 68 | 1.58 |
| *CSF1R* | 36 | 3.57 | 146 | 3.39 |
| *NOTCH3* | 58 | 5.75 | 234 | 5.44 |

**Table S7**. **Relative frequency of total variants in the 10 adult-onset leukodystrophy genes in our discovery cohort and EVS**. Freq., frequency; EVS, exome variant server; n, number; tot, total.

| **Gene** | **Transcript** | **Position** | **N. variants** | **Test** | **P-value** | **Adj-p-value** |
| --- | --- | --- | --- | --- | --- | --- |
| *NOTCH3* | NM_000435 | chr19:15270477..15303314 | 83 | CALPHA | 0,00125707 | 0,0125707 |
| *TREX1* | NM_016381 | chr3:48506936..48509036 | 19 | CALPHA | 0,0560345 | 0,560345 |
| *EIF2B2* | NM_014239 | chr14:75469634..75476279 | 14 | CALPHA | 0,107438 | 1 |
| *CSF1R* | NM_005211 | chr5:149432863..149466105 | 47 | CALPHA | 0,391304 | 1 |
| *EIF2B4* | NM_001034116 | chr2:27587266..27593297 | 15 | CALPHA | 0,666667 | 1 |
| *EIF2B5* | NM_003907 | chr3:183852861..183862859 | 24 | CALPHA | 0,714286 | 1 |
| *EIF2B3* | NM_001261418 | chr1:45316209..45452227 | 18 | CALPHA | 0,833333 | 1 |
| *HTRA1* | NM_002775 | chr10:124249093..124274340 | 13 | CALPHA | 0,833333 | 1 |
| *EIF2B1* | NM_001414 | chr12:124105572..124118308 | 28 | CALPHA | 1 | 1 |
| *ARSA* | NM_000487 | chr22:51063470..51066552 | 32 | CALPHA | 1 | 1 |

**Table S8a**. **Results from the CALPHA test performed**. Position is in hg19/GRCh37. Adj- p-value, adjusted p-value after Bonferroni correction (p-value*10 [number of genes considered in the single-gene based analysis]). N, number.

| **Gene** | **Transcript** | **Position** | **N. variants** | **Test** | **P-value** | **Adj-p-value** |
| --- | --- | --- | --- | --- | --- | --- |
| *EIF2B1* | NM_001414 | chr12:124105572..124118308 | 24 | SKAT | 0,125412 | 1 |
| *ARSA* | NM_000487 | chr22:51063470..51066552 | 32 | SKAT | 0,181769 | 1 |
| *EIF2B5* | NM_003907 | chr3:183852977..183862859 | 22 | SKAT | 0,247673 | 1 |
| *EIF2B4* | NM_001034116 | chr2:27587266..27593297 | 15 | SKAT | 0,345016 | 1 |
| *TREX1* | NM_016381 | chr3:48506936..48509036 | 17 | SKAT | 0,479248 | 1 |
| *CSF1R* | NM_005211 | chr5:149432917..149466105 | 41 | SKAT | 0,69913 | 1 |
| *EIF2B3* | NM_001261418 | chr1:45316209..45452181 | 15 | SKAT | 0,721593 | 1 |
| *EIF2B2* | NM_014239 | chr14:75469634..75476279 | 13 | SKAT | 0,738056 | 1 |
| *NOTCH3* | NM_000435 | chr19:15270477..15303314 | 72 | SKAT | 0,849156 | 1 |
| *HTRA1* | NM_002775 | chr10:124249093..124274340 | 12 | SKAT | 0,854183 | 1 |

**Table S8b**. **Results from the SKAT test performed**. Position is in hg19/GRCh37. Adj- p-value, adjusted p-value after Bonferroni correction (p-value*10 [number of genes considered in the single-gene based analysis]).

| Gene | Position | cDNA | Aa  change | ExAc | EVS | AD  Carriers (tot=332) | CTRLS  Carrier  (tot=676) | MT | P-value | Adj P-value_§_ | OR | CI |
| --- | --- | --- | --- | --- | --- | --- | --- | --- | --- | --- | --- | --- |
| *NOTCH3* | chr19:15285052 | c.A4563G | p.P1521P | 0,881 | 0,1 | 134 | 188 | disease_causing | 7,521e-05 | 0.02 | 1,755 | 1,31-2,33 |
| *TREX1* | chr3:48507667** | UTR5 | UTR5 | NA | NA | 76 | 205 | NA | 0.0137 | 1 | 0,68 | 0,49-0,93 |
| *TREX1* | chr3:48508585** | c.C696T | p.Y232Y | 0,435 | 0,453 | 212 | 480 | polymorphism | 0.02506 | 1 | 0,72 | 0,54-0,96 |
| *NOTCH3* | chr19:15270929* | UTR3 | UTR3 | NA | NA | 7 | 4 | NA | 0.04741 | 1 | 3,61 | 0,91-16,95 |
| *EIF2B5* | chr3:183860063 | c.G1341A | p.S447S | 0,00781 | 0,006512 | 2 | 16 | disease_causing | 0.07264 | 1 | 0,25 | 0,027-1,074 |
| *EIF2B5* | chr3:183861243 | c.A1759G | p.I587V | 0,3138 | 0,328 | 193 | 353 | polymorphism | 0.08059 | 1 | 1,27 | 0,966-1,67 |
| *EIF2B1* | chr12:124118248*** | UTR5 | UTR5 | NA | NA | 18 | 21 | NA | 0.0828 | 1 | 1,78 | 0,88-3,57 |
| *NOTCH3* | chr19:15271771* | c.C6668T | p.A2223V | 0,7591 | 0,198 | 107 | 255 | polymorphism | 0.09381 | 1 | 0,785 | 0,58-1,04 |
| *EIF2B1* | chr12:124106254*** | UTR3 | UTR3 | NA | NA | 4 | 2 | NA | 0.09562 | 1 | 4,1 | 0,58-45,57 |

**Table S9.** Position is in hg19/GRCh37. MAF, minor allele frequency; */**/*** define variants that cluster within the same haplotype block. Adj P-value, adjusted P-value, based on 215 variants detected. Aa, amino acid; CTRLS, controls, OR, odds ratio; CI, confidence interval.

| Gene | Transcript | KBPs of coding sequence | Coding non syn low-freq-rare | Frequency of low-freq-rare missense coding variants |
| --- | --- | --- | --- | --- |
| *EIF2B4* | NM_001034116 | 1.563 | 2 | 1.28 |
| *TREX1* | NM_016381 | 1.11 | 2 | 1.8 |
| *HTRA1* | NM_002775 | 1.443 | 3 | 2.08 |
| *EIF2B1* | NM_001414 | 0.918 | 2 | 2.18 |
| *EIF2B5* | NM_003907 | 2.166 | 6 | 2.77 |
| *NOTCH3* | NM_000435 | 6.966 | 23 | 3.301 |
| *EIF2B2* | NM_014239 | 1.056 | 4 | 3.78 |
| *EIF2B3* | NM_001261418 | 1.206 | 5 | 4.14 |
| *ARSA* | NM_000487 | 1.53 | 7 | 4.57 |
| *CSF1R* | NM_005211 | 2.919 | 16 | 5.48 |

**Table S11. Relative frequency of low frequency and rare coding variants in the 10 adult-onset leukodystrophy genes studied.** Freq., frequency; kbps, kilobase pairs. Syn, synonymous.

| **Gene** | **cDNA change** | **Aa change** | **pathogenicity** |
| --- | --- | --- | --- |
| ***PSEN1*** | c.313T>A,G; c.314T>G;c.315T>G | p.F**105**I, V; C; L | pathogenic, 1 family, 1 family; 1 family; 1 family |
|  | c.338T>A; C | p.L**113**Q; P | pathogenic, 1 patient Alzheimer Disease / Myoclonus (Autopsy Proven); 1 family, segregation proven |
|  | c.343T>C, C; c.344A>G | p.Y**115**H, D; C | pathogenic, 3 families; 1 family; 5 families |
|  | c.347C>A; T | p.T**116**N; I | pathogenic, 4 families; 3 families |
|  | c.349C>G, T; c.350C>G, T | p.P**117**A, S; R, L | pathogenic, 2 families, 1 family; 2 families, 2 families |
|  | c.358G>A; c.359A>G | p.E**120**K; G | pathogenic, 2 families; pathogenic, 1 family |
|  | c.360A>C; T | p.E**120**D; D | pathogenic, 3 families; 1 family |
|  | c.403A>G; c.404A>G | p.N**135**D; S | pathogenic, 1 family; 2 families |
|  | c.415A>G; c.416T>A, C; c.417G>A, C | p.M**139**V; K, T;I,I | pathogenic, 9 families; 2 families, 6 families; 1 family, 1 family |
|  | c.427A>G, T; c.428T>A, C; c.429T>G | p.I**143**V, F; N, T;M | pathogenic, 1 family; 1 family; 1 family, 8 families; 1 family |
|  | c.436A>C, G, T; c.438G>A, C, T | p.M**146**L, V, L;I, I,I | pathogenic, 15 families; 4 families, 2 families; 2 families, 1 family, 1 family |
|  | c.439A>C; c.440C>T | p.T**147**P; I | pathogenic, 1 family; 1 family |
|  | c.460T>A; G | p.Y**154**N; C | pathogenic, 1 family |
|  | c.487C>T; c.488A>C, G | p.H**163**Y; P, R | pathogenic, 1 family; 1 family, 22 families |
|  | c.493T>G; c.495G>C | p.W**165**G; C | pathogenic, 1 family; 1 family |
|  | c.497T>A; C; G | p.L**166**H; P; R | pathogenic, 1 family; 1 family; 1 family |
|  | c.505T>C; c.506C>T | p.S**169**P; L | pathogenic, 1 family; 2 families |
|  | c.518T>G; c.519G>C, T | p.L**173**W; F, F | pathogenic, 1 family; 1 family, 1 family |
|  | c.520C>A | p.L**174**M | pathogenic, 2 families |
|  | c.529T>C; c.530T>C | p.F**177**L; S | pathogenic, 2 family; 1 family |
|  | c.551A>G; C | p.E**184**G; D | pathogenic, 2 families; 2 families |
|  | c.616G>A; c.617G>A, C, T | p.G**206**S; D, A, V | pathogenic, 3 families; 2 families, 19 families, 1 family |
|  | c.625G>A; c.626G>A, T | p.G**209**R; E, V | pathogenic, 1 family; 1 family, 1 family |
|  | c.637A>C, T; c.638T>C | p.I**213**L, F; T | pathogenic, 1 family, 1 family; 1 family |
|  | c.640C>G; T | p.H**214**D; Y | pathogenic, 1 family; 1 family |
|  | c.649G>C; A | p.G**217**R; D | pathogenic, 1 family; 2 families |
|  | c.655C>T; c.656T>C, G | p.L**219**F; P, R | pathogenic, 1 family; 1 family, 1 family |
|  | c.665A>G; c.666G>C | p.Q**222**R; H | pathogenic, 1 family; 2 families |
|  | c.676C>T; c.677T>G | p.L**226**F; R | pathogenic, 2 families; 1 family |
|  | c.689G>T; c.690T>G | p.S**230**I; R | pathogenic, 1 family; 1 family |
|  | c.691G>A; T | p.A**231**T; V | pathogenic, 2 families; 1 family |
|  | c.697A>C,G, T; c.698T>C; c.699G>A, C | p.M**233**L, V, L; T; I, I | pathogenic, 4 families; 1 family; 6 families; 1 family, 1 family |
|  | c.703C>G; c.704T>C, G | p.L**235**V; P, R | pathogenic, 1 family; 3 families, 1 family |
|  | c.709T>A; C | p.F**237**I; L | pathogenic, 1 family; 1 family |
|  | c.743T>C; G | p.L**248**P; R | pathogenic, 1 family; 1 family |
|  | c.748T>G; c.749T>C | p.L**250**V; S | pathogenic 2 families; 1 family |
|  | c.781G>C; T | p.V**261**L; F | pathogenic, 1 family; 5 families |
|  | c.787T>C; c.788G>T | p.C**263**R; F | pathogenic, 1 family; 2 families |
|  | c.799C>T; c.800C>T | p.P**267**S; L | pathogenic, 1 family; 1 family |
|  | c.805C>G | p.R**269**G | pathogenic 2 families |
|  | c.806G>A | p.R**269**H | pathogenic 4 families |
|  | c.818A>C; G | p.E**273**A; G | pathogenic, 1 family; 1 family |
|  | c.833G>A, C, T; c.834A>C | p.R**278**K, T, I; S | pathogenic, 1 family, 1 family, 1 family; 1 family |
|  | c.839A>C, G | p.E**280**A, G | pathogenic 12 families, 6 families |
|  | c.844C>G, T; c.845T>G | p.L**282**V, F; R | pathogenic, 1 family, 1 family; 2 families |
|  | c.850C>T; c.851C>T | p.P**284**S; L | pathogenic, 1 family; 1 family |
|  | c.856C>G; c.857T>C | p.L**286**V; P | pathogenic 3 families; 1 family |
|  | c.1129A>T; c.1130G>T | p.R**377**W; M | pathogenic 2 families; 1 family |
|  | c.1133G>A, T | p.G**378**E, V | pathogenic 4 families, 1 family |
|  | c.1141C>G | p.L**381**V | pathogenic 2 families |
|  | c.1157T>C; c.1158C>A | p.F**386**S, L | pathogenic, 1 family, 1 family |
|  | c.1174C>G; c.1175T>C | p.L**392**V; P | pathogenic 6 families, 1 family |
|  | c.1270C>G, T; c.1271T>A, G | p.L**424**F, F; H, R | pathogenic, 1 family, 1 family; 2 families, 1 family |
|  | c.1292C>A, T | p.A**431**E, V | pathogenic 34 families, 1 family |
|  | c.1300G>A, T; 1301C>G | p.A**434**T; C | pathogenic, 1 family; 2 families |
|  | c.1306C>T; c.1307C>A | p.P**936**S; Q | pathogenic, 1 family; 3 families |
| ***PSEN2*** | c.364A>C; c.365C>G | p.T**122**P; R | pathogenic 2 families; 1 family |
|  | c.421A>T; c.422A>T | p.N**141**Y; I | pathogenic, 1 family; 10 families |
|  | c.715A>G; c.717G>A | p.M**239**V; I | pathogenic 6 families; 1 family |
| ***APP*** | c.2077G>A, C; c.2078A>G | p.E**693**K, Q; G | pathogenic 3 families, 4 families; 2 families |
|  | c.2140A>G; c.2141C>T | p.T**714**A; I | pathogenic 3 families; 3 families |
|  | c.2143G>A; c.2144T>C | p.V**715**M; A | pathogenic 2 families; 4 families |
|  | c.2146A>G, T; c.2147T>C; c.2148C>G | p.I**716**V, F; T; M | pathogenic 1 family; 1 family; 1 family; 1 family |
|  | c.2149G>A, C, T; c.2150T>G | p.V**717**I, L, F; G | pathogenic 38 families, 7 families, 3 families; 2 families |
| ***MAPT*** | c.14G>A, T | p.R**5**H, L | pathogenic 1 family, 1 family |
|  | c.1906C>A,T; c.1907C>T | p.P**636**T, S; L | pathogenic 1 family, 2 familes; 32 familes |
|  | c.1919G>A, T | p.S**640**N, I | pathogenic 3 familes; 1 family |
|  | c.2008G>A; c.2009G>T | p.G**670**S; p.G**670**V | pathogenic 1 family; 1 family |
|  | c.2170G>A, C | p.G**724**R,R | pathogenic 2 familes; 2 familes |

**Table S12*. PSEN1*, *PSEN2, APP* and *MAPT* missense mutations within the same highly conserved codon always lead to a pathogenic phenotype (AD and FTD spectrum). These data are taken from http://www.molgen.ua.ac.be/ADMutations/.**

| Patient | *CSF1R* TK mutation | AO-AD | Family history of dementia | disease duration | First symptom | Behavioural symptom | Motor symp | Other sign-symptoms | Misdiagnosis | CT/MRI | Neuropath | References |
| --- | --- | --- | --- | --- | --- | --- | --- | --- | --- | --- | --- | --- |
| II-1 | p.E633K | 78-84 | + | 6y | Cognitive decline | Psychiatric syndrome | Parkinsonism | Seizures, syncopal episodes | Probable AD | Cortical atrophy | Frontal atrophy, myelin loss, cortical axonal spheroids, senile plaques in cortex and hippocampus, amyloid angiopathy, neurofibrillary tangles | (Baba et al., 2006)  (Rademakers et al., 2011) |
| II-4 | p.E633K | 54-62 | + | 8y | Atypical Parkinsonism | depression | Atypical Parkinsonism |  | CBD | Bilateral high intensity lesions cortical WM | Cortical atrophy, axonal spheroids, cortical senile plaques and amyloid angiopathy, neurofibrillary tangles | (Baba et al., 2006)  (Rademakers et al., 2011) |
| Sporadic patient | NA | 32-49 | - | 7y | Personality changes, memory impairment |  | Increasing motor impairment with rigidity | seizures |  | Symmetrical periventricular parietal white matter hyperintensities | Severe myelin loss in the frontal subcortical WM, subcortical amyloid angiography, axonal spheroids | (Browne et al., 2003) |

**Table S13. Features of HDLS patients with AD neuropathology.** AO-AD, age at onset-age at death; symp, symptom; CBD, corticobasal degeneration; NA, not available.

| **Gene** | **Forward Primer** | **Reverse Primer** |
| --- | --- | --- |
| *Csf1r* | ATCACTGACCCTGCACTGAA | TTCACCATGGTCTTGCACAC |
| *Arsa* | ACCCTCAGTTCAGTGGACAA | TCTGCAGTGAAGATGACTAGTGT |
| *Eif2b1* | TTTACGGACCTGGGTGTGTT | TGGATTCAGTTTCAGCCCCT |
| *Eif2b2* | GAGCACATCCACTCCAACGA | TCTGCAACAATAACGTGGAACT |
| *Eif2b3* | AGACGACACTCTGACCTTGG | GGCTTCCATGTAGAGTCCCA |
| *Eif2b4* | TGGATCTGTGATGTCTCGGG | GTCTGCACACGTTCACAGAA |
| *Eif2b5* | AAGTGGTGCCATCCTACGTC | AGGGCTCTGCAGATATTGATATT |
| *Notch3* | AGATGTGGATGAGTGTCAGC | AGGCACAGTAGAAAGAGGCC |
| *Htra1* | CAGAGCTGAGACCTGGAGAA | GCGTCTGTCTGAATGTAGTCC |
| *Trex1* | AACGCTTCGATGACAACCTG | TCCTCGAACCATTCCCTGAG |
| *Il-34* | TGGTGAGTCTCAATGCCACT | GCTGTACGTTCTCCAGCAAT |
| *Csf1* | CACAACCTCATCCTTCTGCG | TGTCAGTCTCTGCCTGGATG |
| *Aif1* | ATGCTGGAGAAACTTGGGGT | GCCCAGCATCATTCTGAGAA |

**Table S14. Sequence of primers used for Real-Time PCR experiments**
